# Supplementary figures and images for: Single-molecule analysis reveals the phosphorylation of FLS2 governs its spatiotemporal dynamics and immunity
Source: eLife. 2024 Jul 24;12:RP91072. doi: 10.7554/eLife.91072 (PMC11268883; doi:10.7554/eLife.91072)

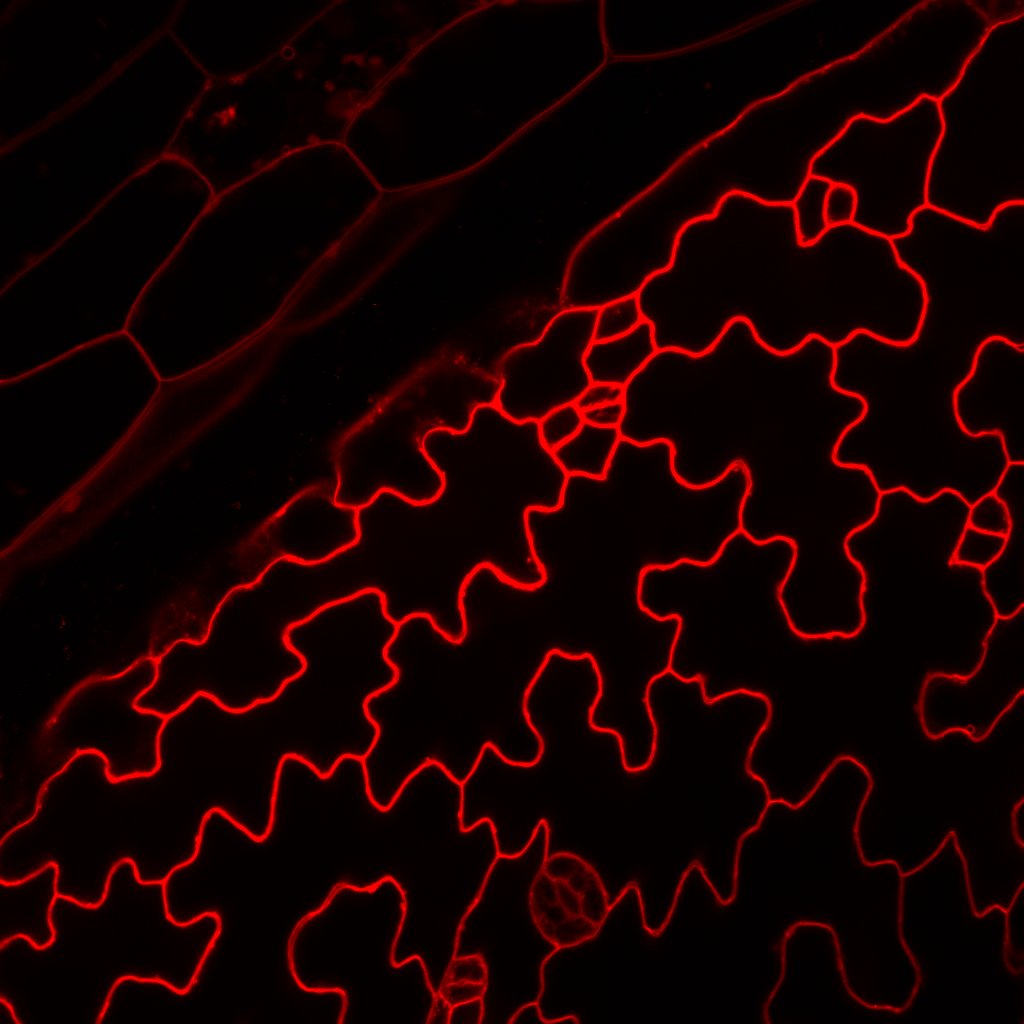

Supplement: Figure 1—figure supplement 1—source data 1. [file elife-91072-fig1-figsupp1-data1.zip › Figure 1 - figure supplement -source data 1/Figure 1 - figure supplement 1---SourceData FLS2-FM4-64.tif]

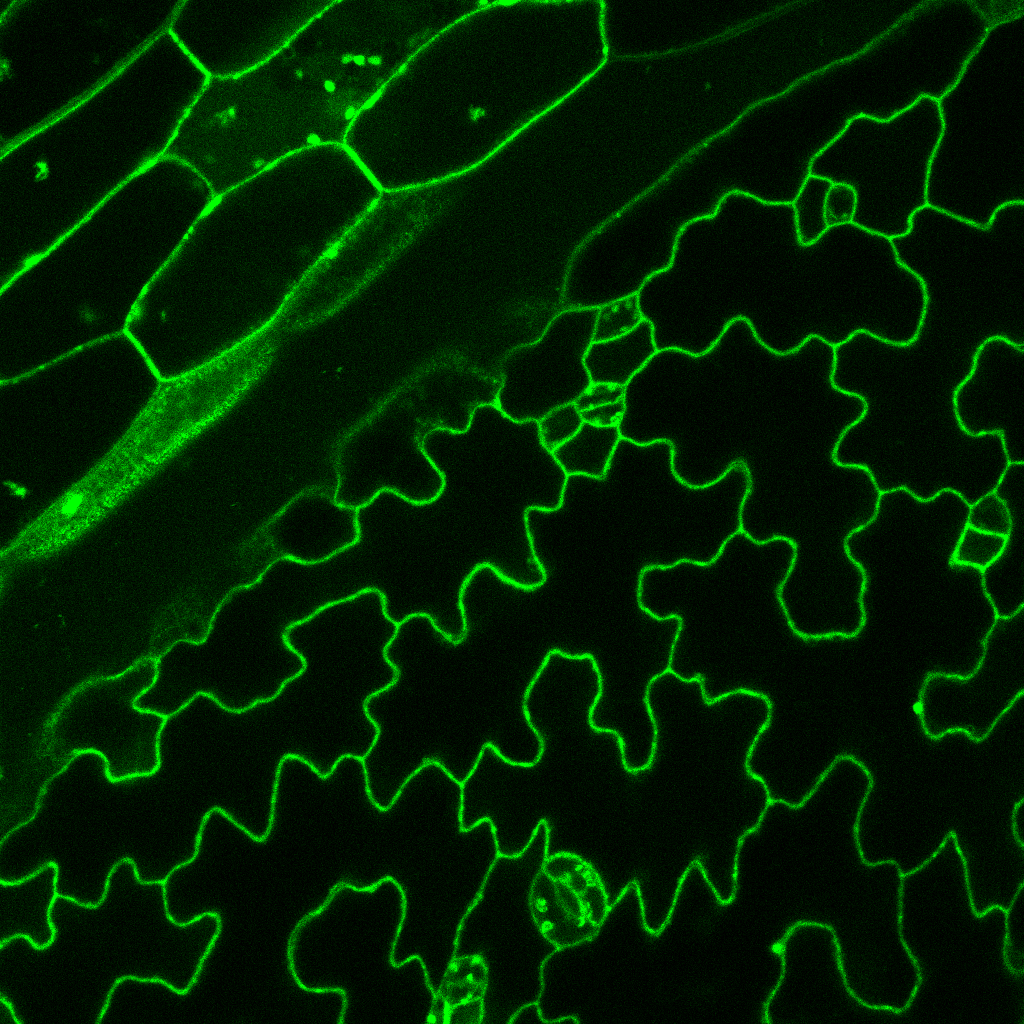

Supplement: Figure 1—figure supplement 1—source data 1. [file elife-91072-fig1-figsupp1-data1.zip › Figure 1 - figure supplement -source data 1/Figure 1 - figure supplement 1---SourceData FLS2-GFP.tif]

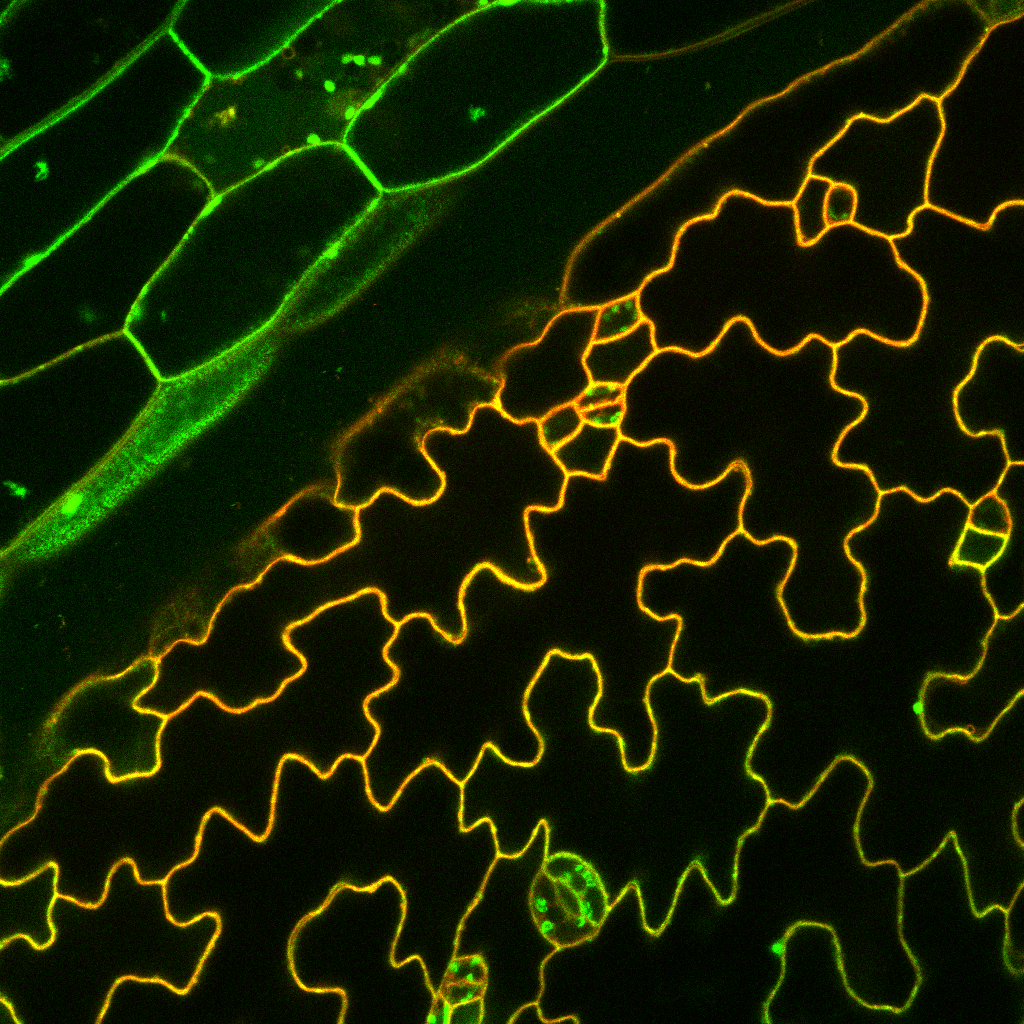

Supplement: Figure 1—figure supplement 1—source data 1. [file elife-91072-fig1-figsupp1-data1.zip › Figure 1 - figure supplement -source data 1/Figure 1 - figure supplement 1---SourceData FLS2-Merge.tif]

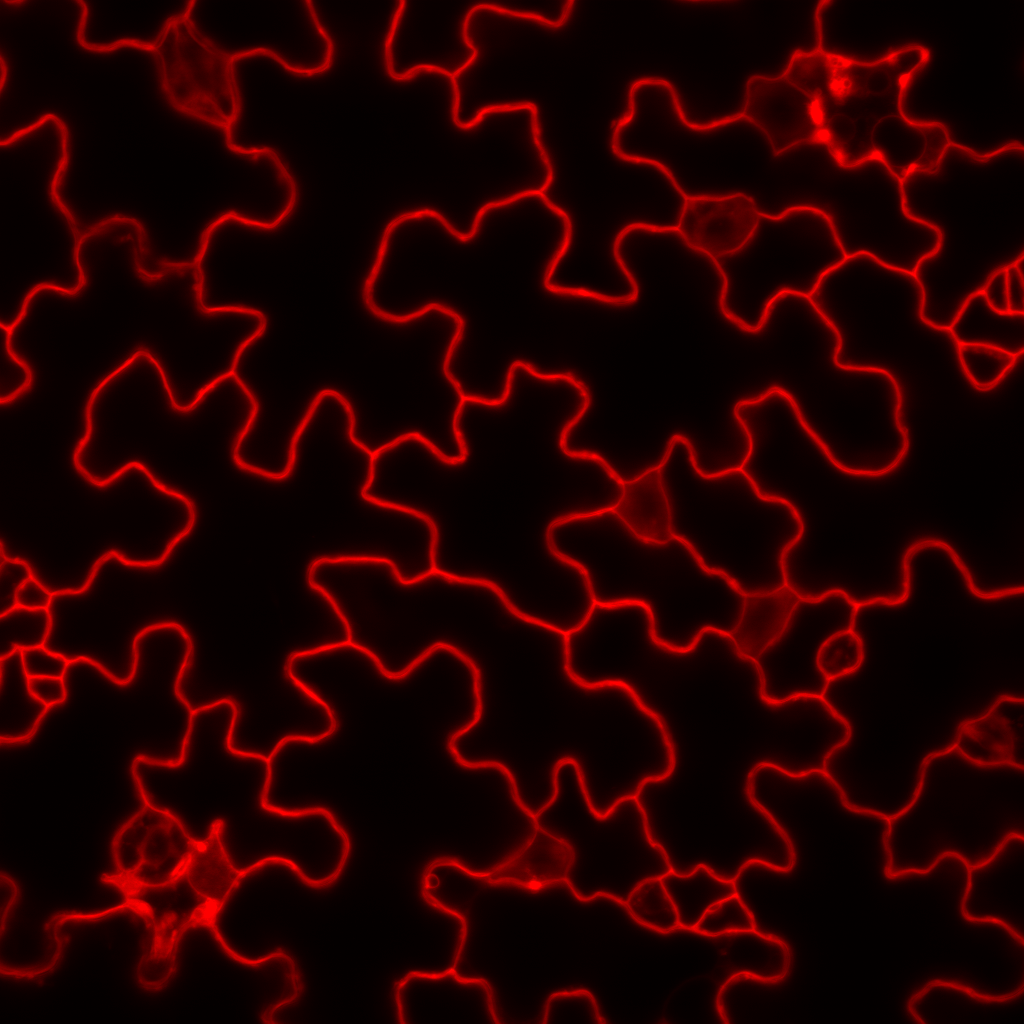

Supplement: Figure 1—figure supplement 1—source data 1. [file elife-91072-fig1-figsupp1-data1.zip › Figure 1 - figure supplement -source data 1/Figure 1 - figure supplement 1---SourceData FLS2S938A-FM4-64.tif]

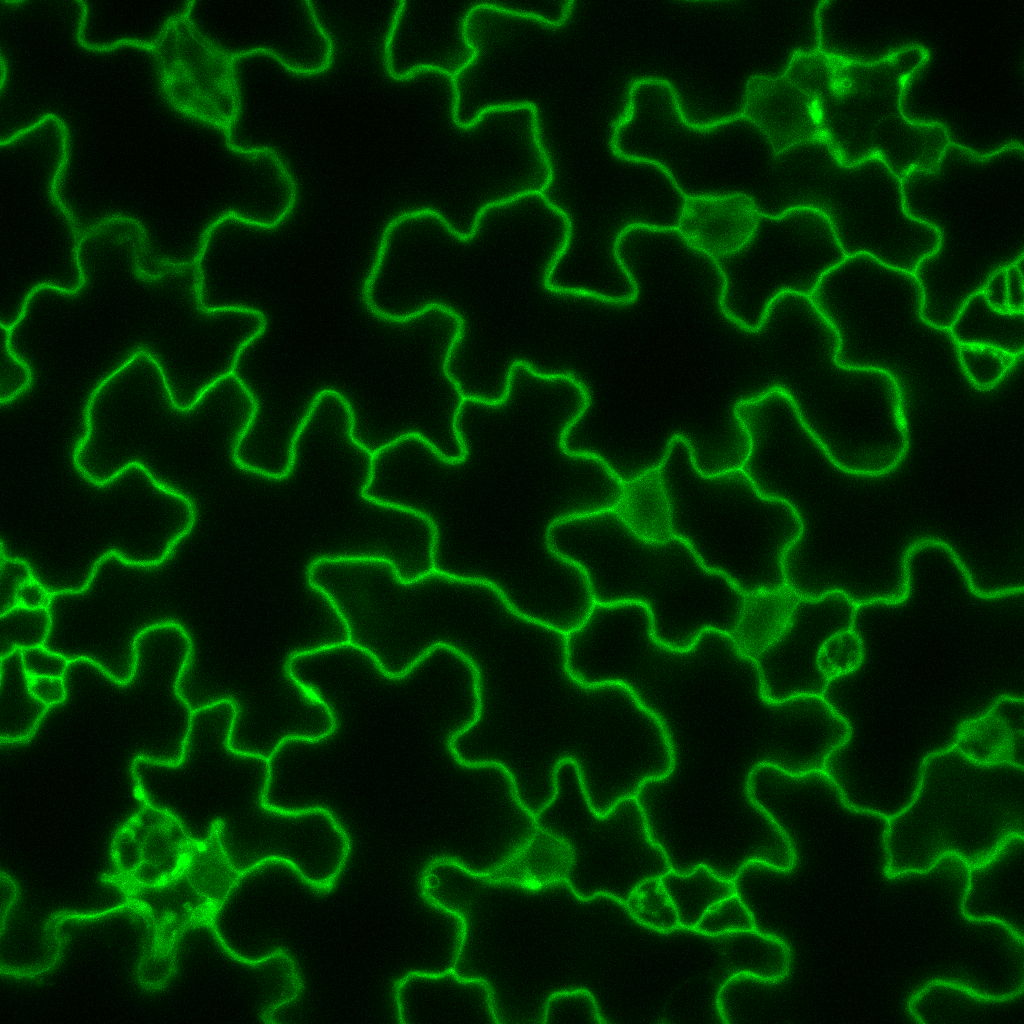

Supplement: Figure 1—figure supplement 1—source data 1. [file elife-91072-fig1-figsupp1-data1.zip › Figure 1 - figure supplement -source data 1/Figure 1 - figure supplement 1---SourceData FLS2S938A-GFP.tif]

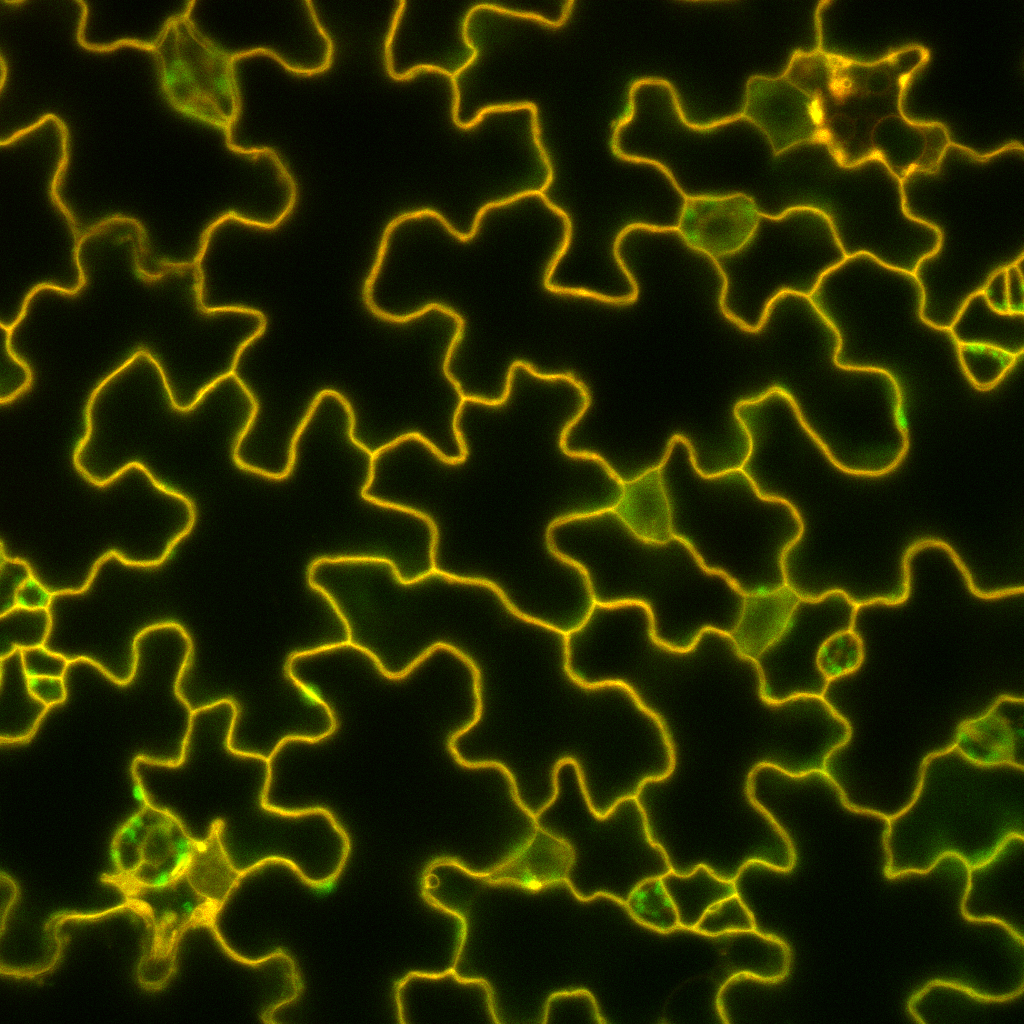

Supplement: Figure 1—figure supplement 1—source data 1. [file elife-91072-fig1-figsupp1-data1.zip › Figure 1 - figure supplement -source data 1/Figure 1 - figure supplement 1---SourceData FLS2S938A-Merge.tif]

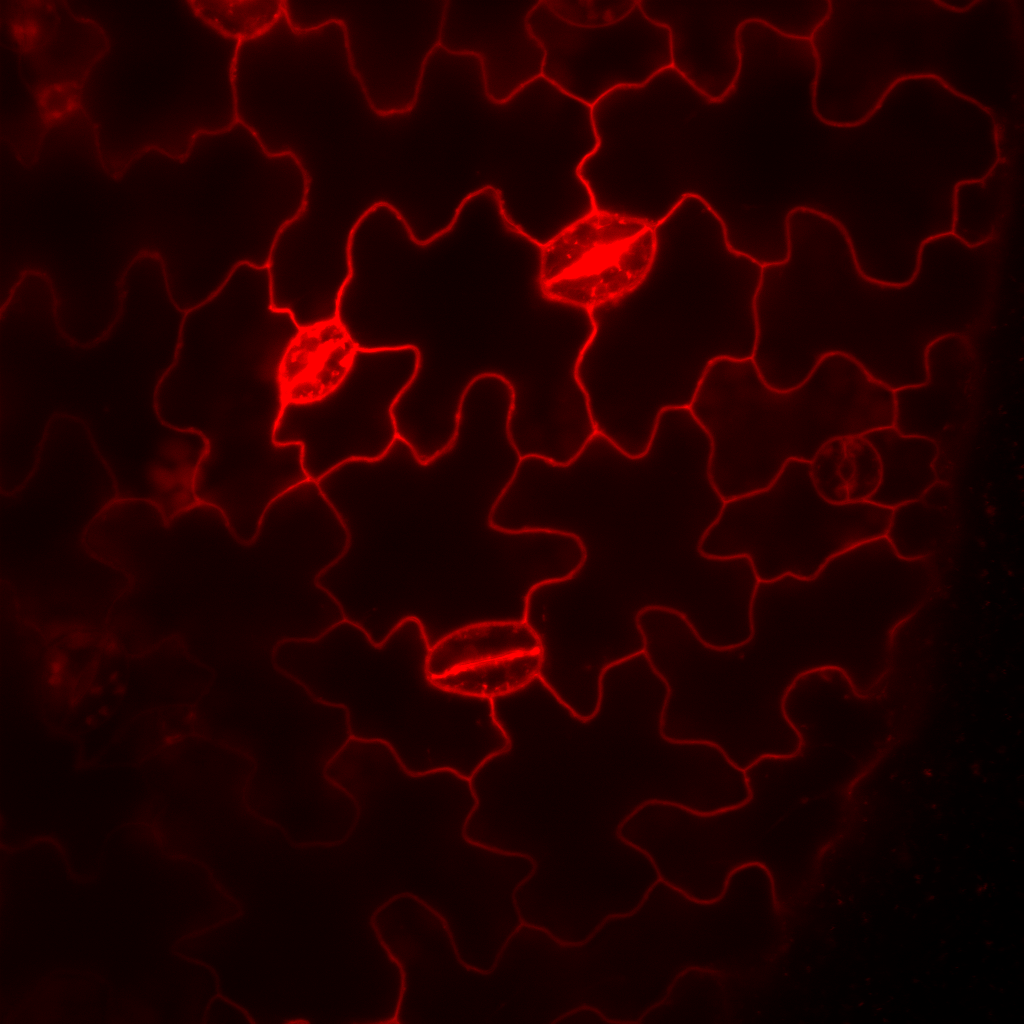

Supplement: Figure 1—figure supplement 1—source data 1. [file elife-91072-fig1-figsupp1-data1.zip › Figure 1 - figure supplement -source data 1/Figure 1 - figure supplement 1---SourceData FLS2S938D-FM4-64.tif]

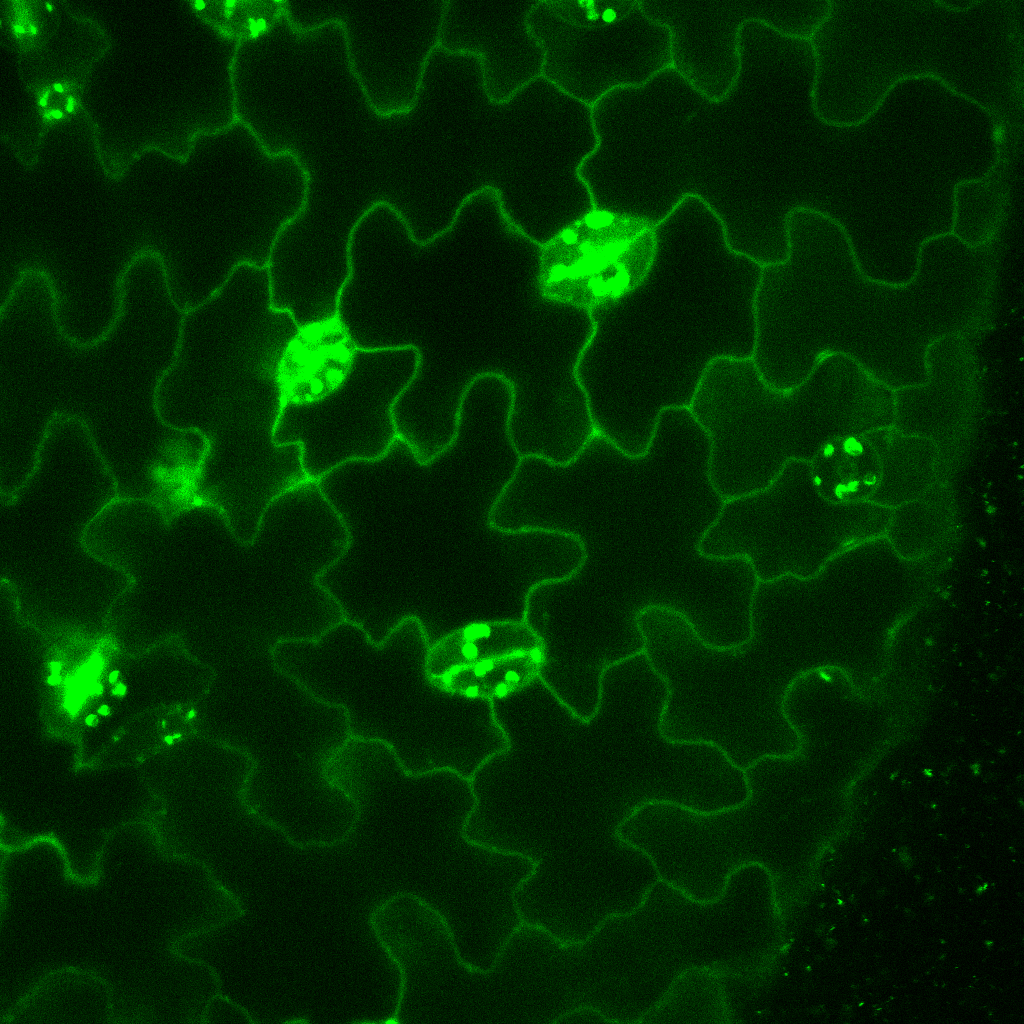

Supplement: Figure 1—figure supplement 1—source data 1. [file elife-91072-fig1-figsupp1-data1.zip › Figure 1 - figure supplement -source data 1/Figure 1 - figure supplement 1---SourceData FLS2S938D-GFP.tif]

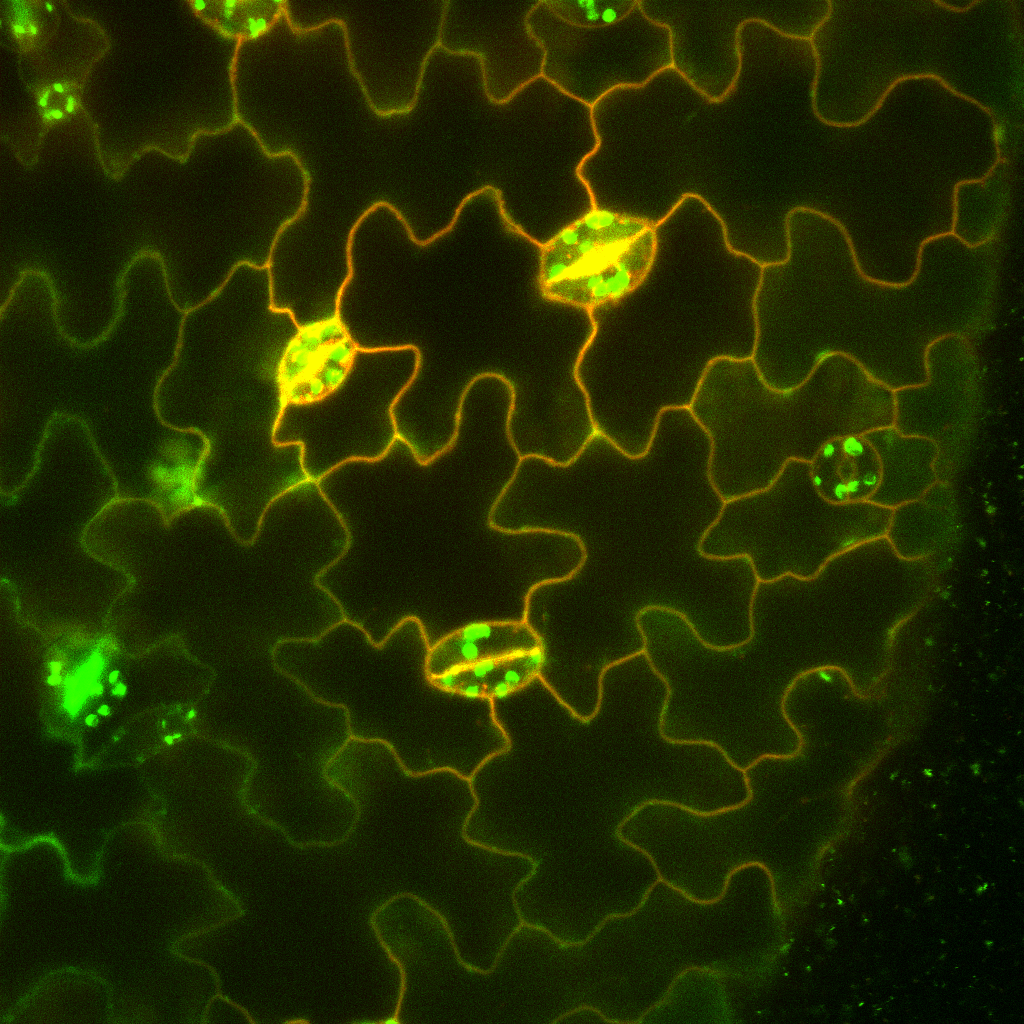

Supplement: Figure 1—figure supplement 1—source data 1. [file elife-91072-fig1-figsupp1-data1.zip › Figure 1 - figure supplement -source data 1/Figure 1 - figure supplement 1---SourceData FLS2S938D-Merge.tif]

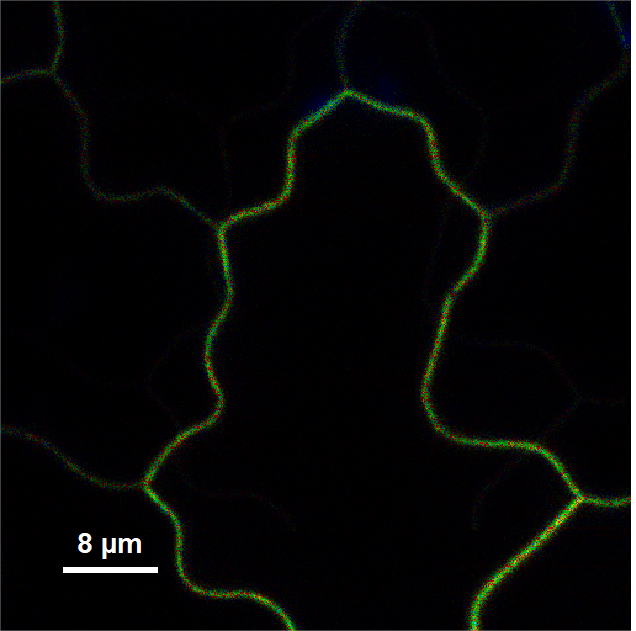

Supplement: Figure 2—source data 4. [file elife-91072-fig2-data4.zip › Figure 2—source data 4/Figure2H---SourceData-FLS2-Rem1.3-CK.bmp]

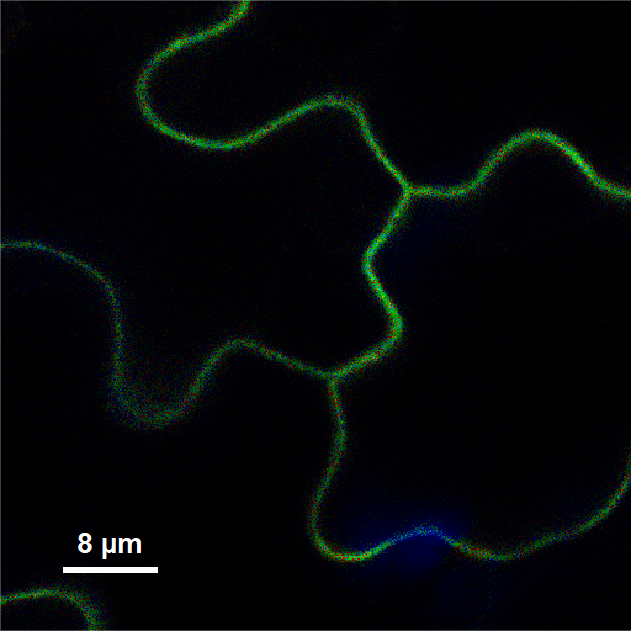

Supplement: Figure 2—source data 4. [file elife-91072-fig2-data4.zip › Figure 2—source data 4/Figure2H---SourceData-FLS2-Rem1.3-flg22.bmp]

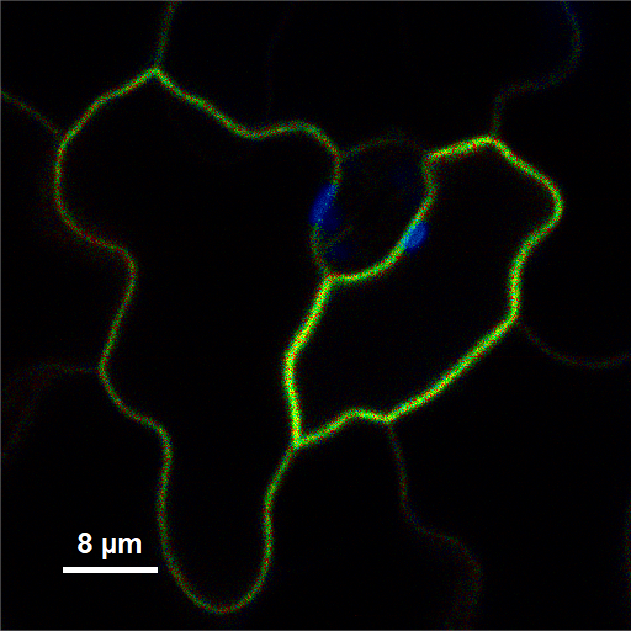

Supplement: Figure 2—source data 4. [file elife-91072-fig2-data4.zip › Figure 2—source data 4/Figure2H---SourceData-FLS2S938A-Rem1.3-CK.bmp]

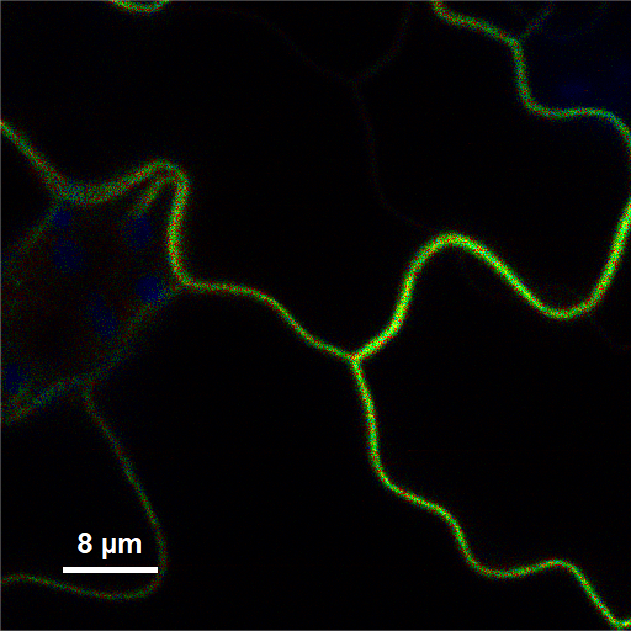

Supplement: Figure 2—source data 4. [file elife-91072-fig2-data4.zip › Figure 2—source data 4/Figure2H---SourceData-FLS2S938A-Rem1.3-flg22.bmp]

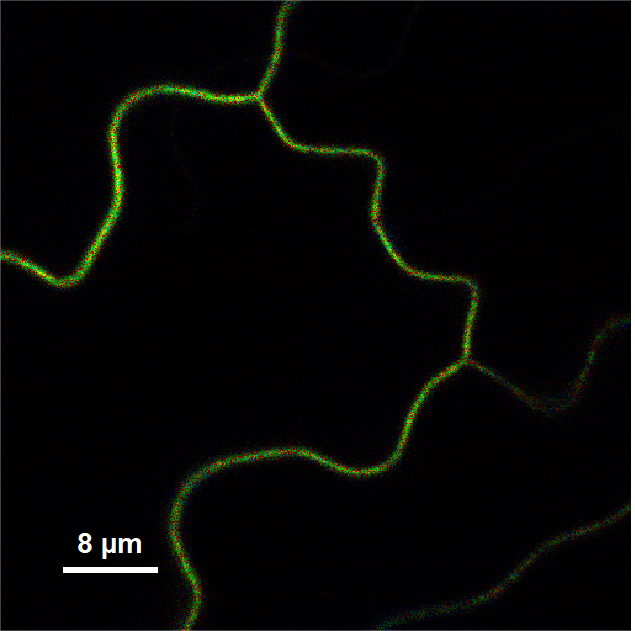

Supplement: Figure 2—source data 4. [file elife-91072-fig2-data4.zip › Figure 2—source data 4/Figure2H---SourceData-FLS2S938D-Rem1.3-CK.bmp]

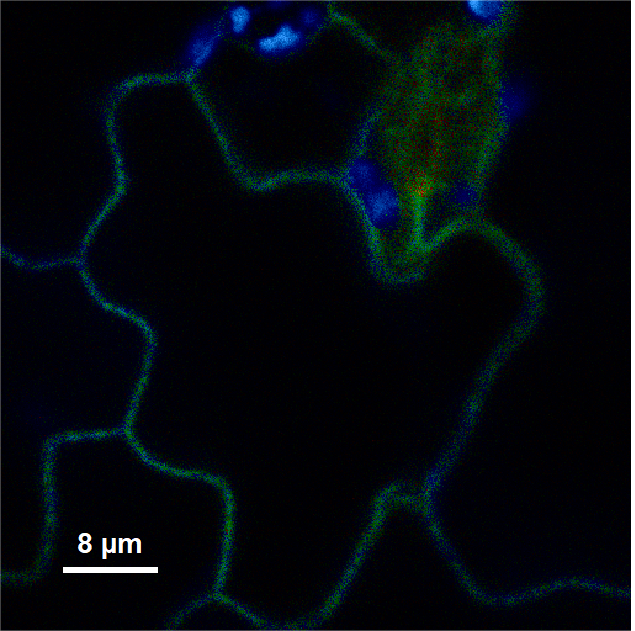

Supplement: Figure 2—source data 4. [file elife-91072-fig2-data4.zip › Figure 2—source data 4/Figure2H---SourceData-FLS2S938D-Rem1.3-flg22.bmp]

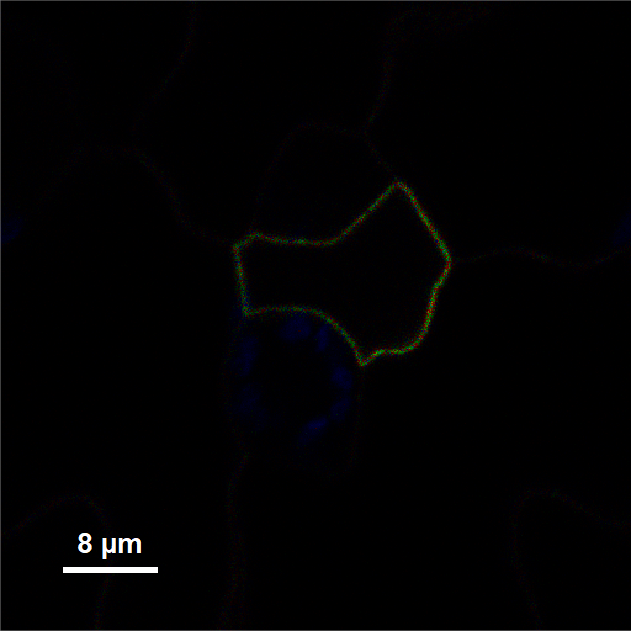

Supplement: Figure 2—source data 4. [file elife-91072-fig2-data4.zip › Figure 2—source data 4/Figure2H---SourceData-GFP.bmp]

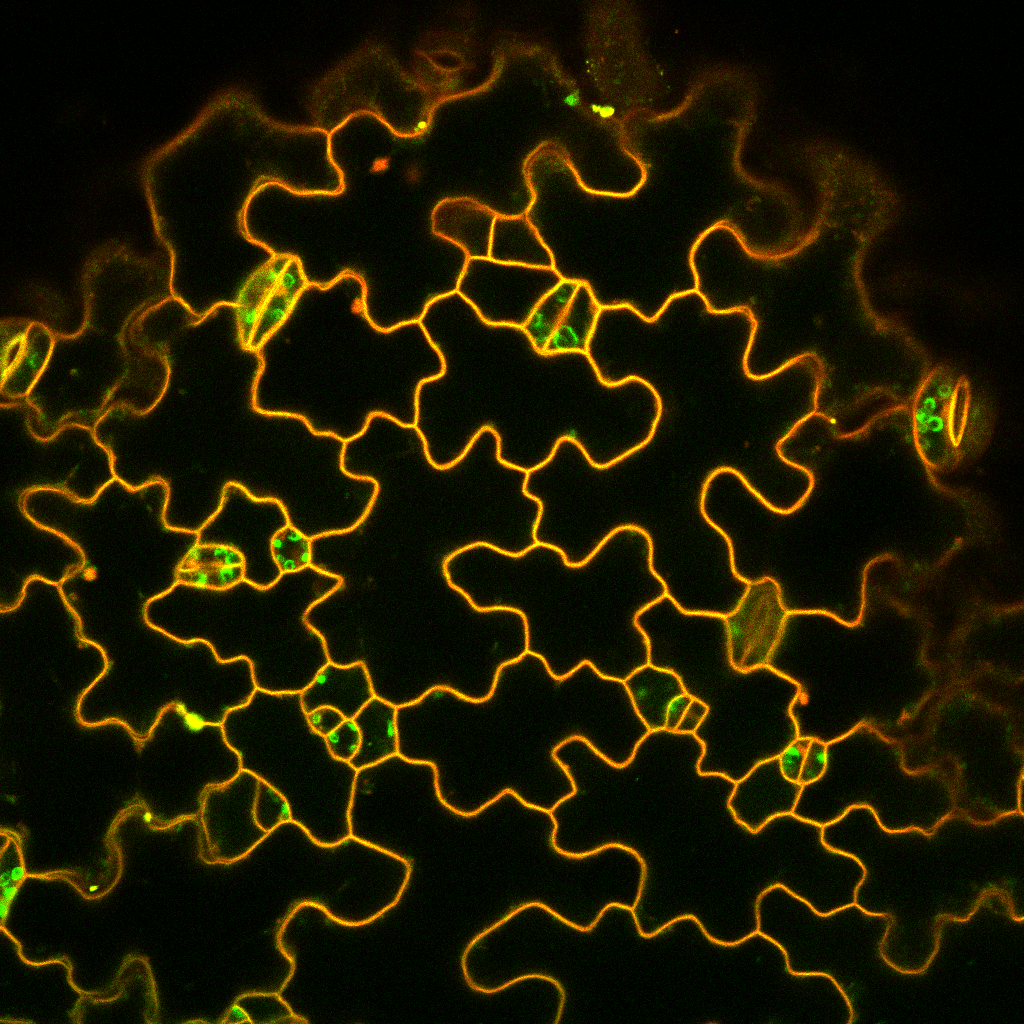

Supplement: Figure 3—source data 1. [file elife-91072-fig3-data1.zip › Figure 3—source data 1/Figure3A---SourceData---FLS2--Merge.tif]

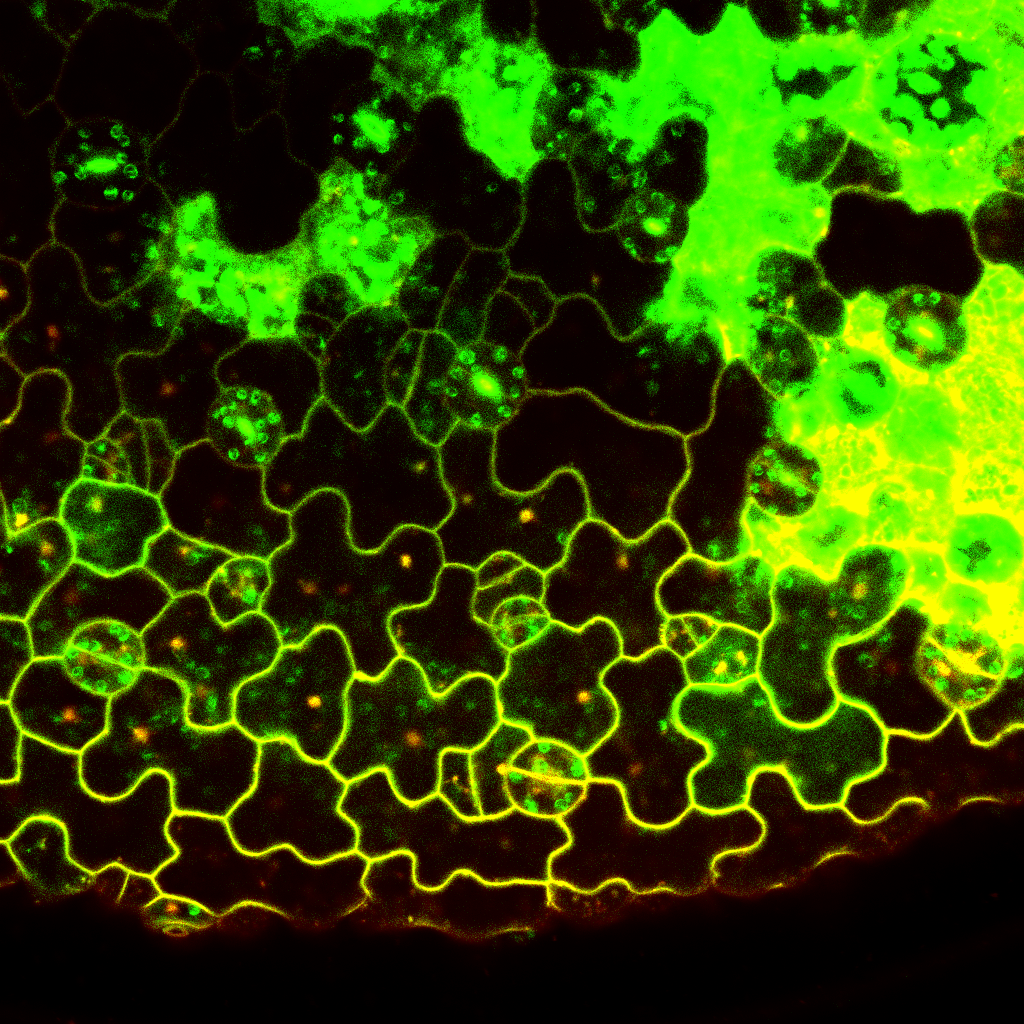

Supplement: Figure 3—source data 1. [file elife-91072-fig3-data1.zip › Figure 3—source data 1/Figure3A---SourceData---FLS2--flg22-Merge.tif]

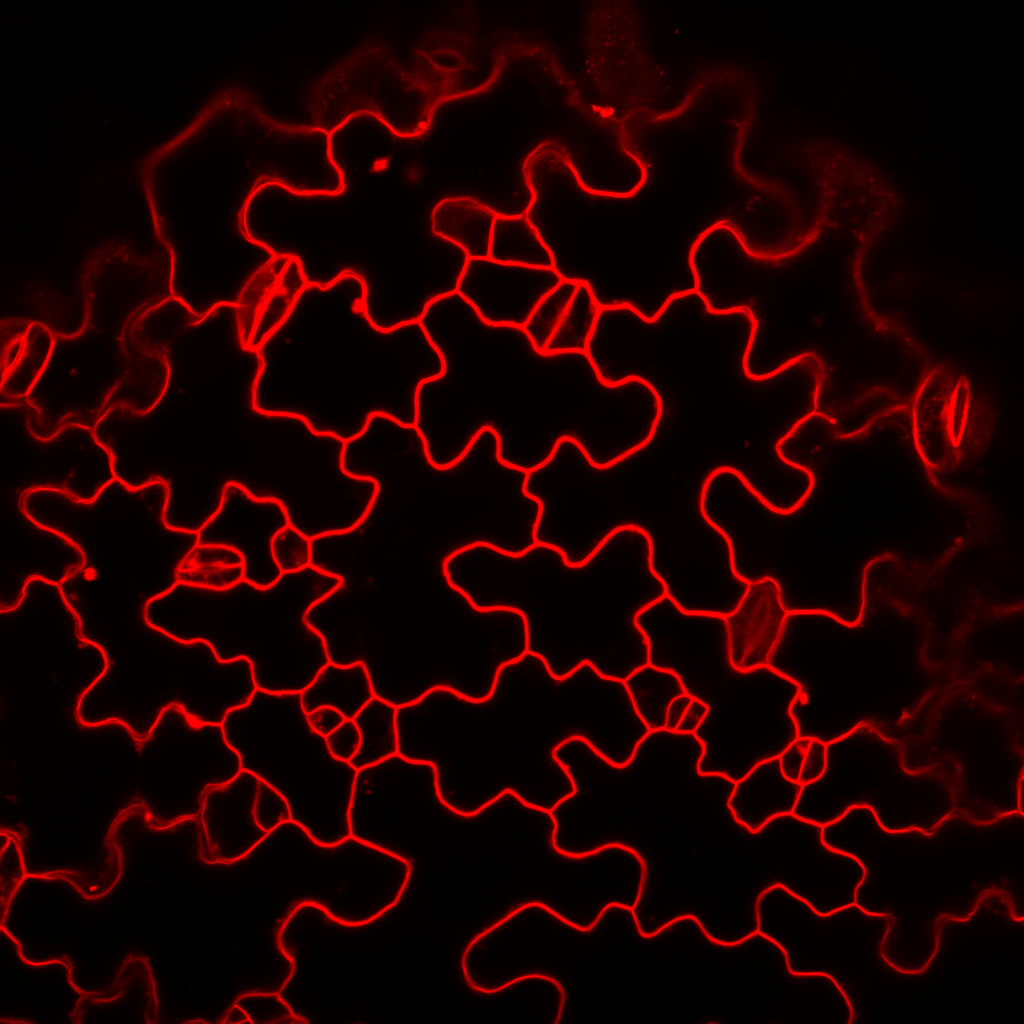

Supplement: Figure 3—source data 1. [file elife-91072-fig3-data1.zip › Figure 3—source data 1/Figure3A---SourceData---FLS2-CHX-BFA-FM4-64.tif]

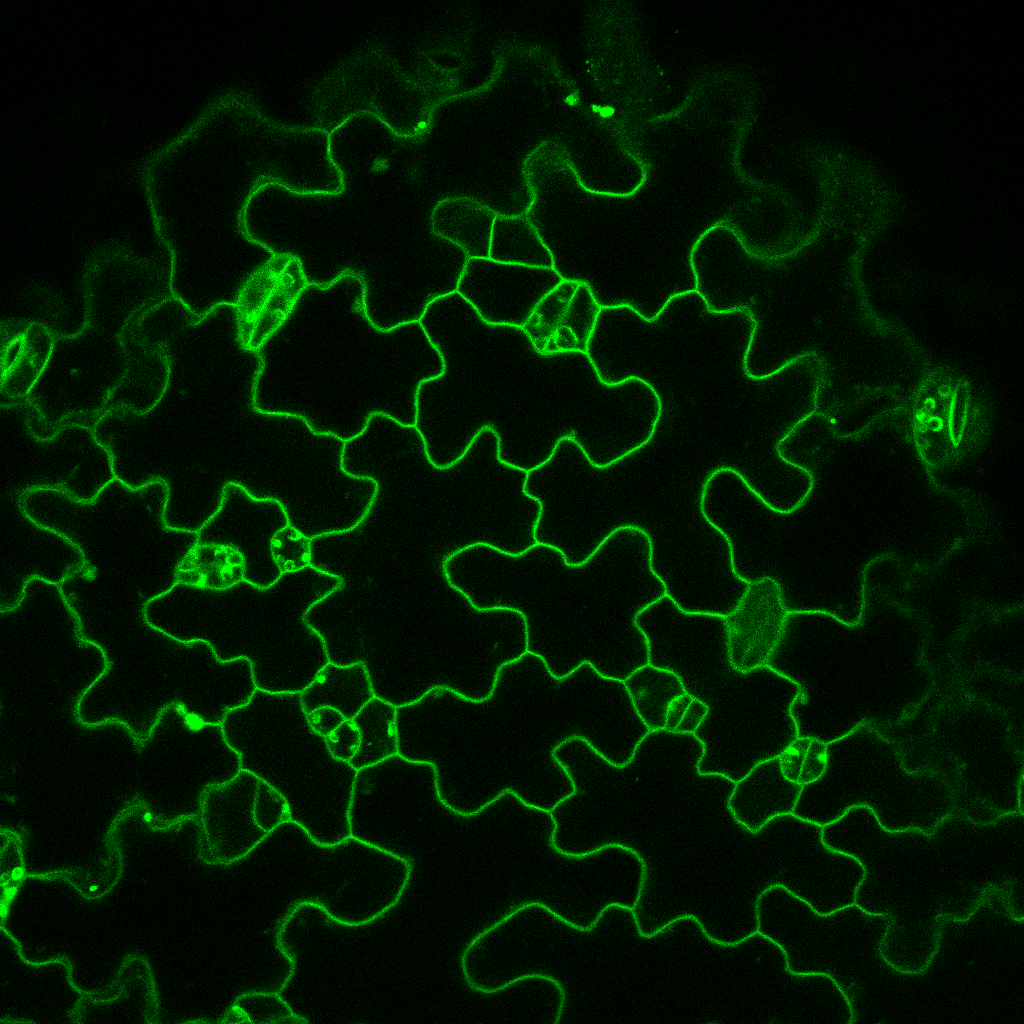

Supplement: Figure 3—source data 1. [file elife-91072-fig3-data1.zip › Figure 3—source data 1/Figure3A---SourceData---FLS2-CHX-BFA.tif]

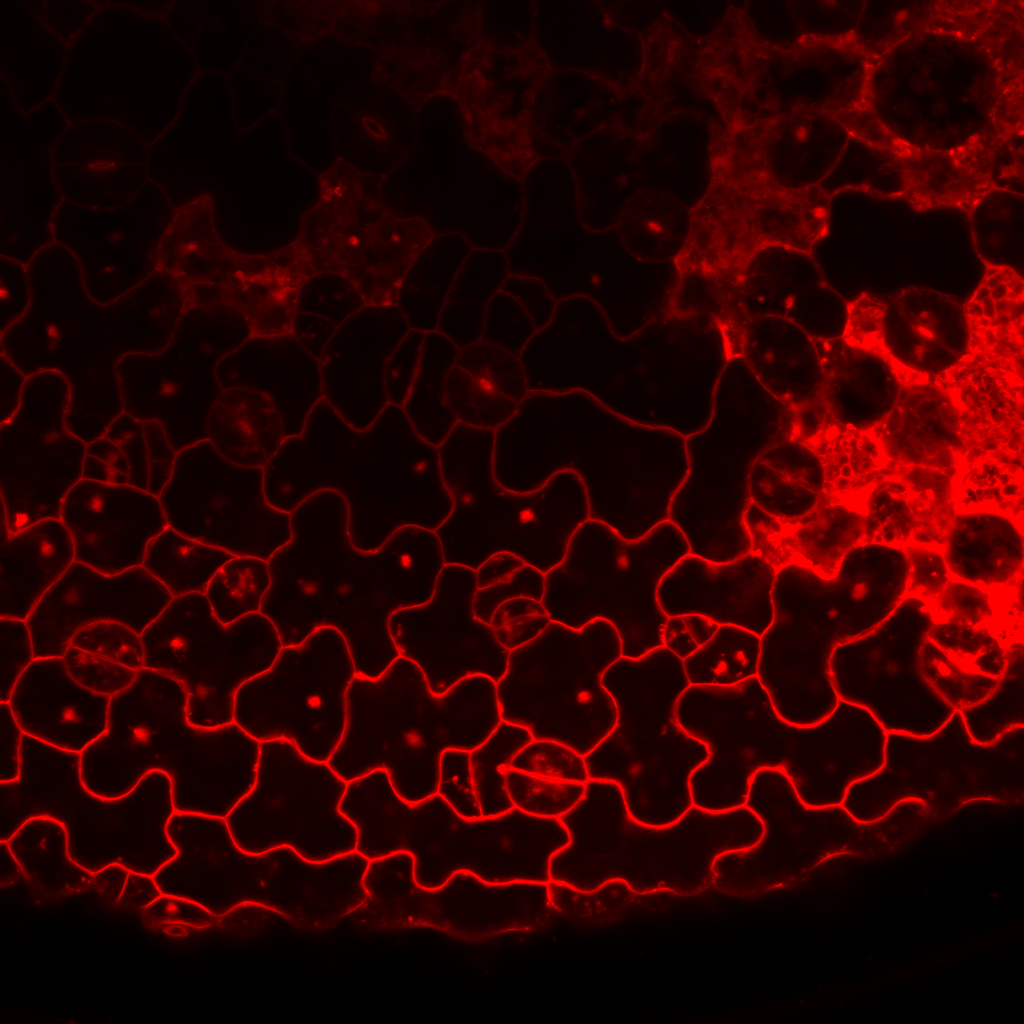

Supplement: Figure 3—source data 1. [file elife-91072-fig3-data1.zip › Figure 3—source data 1/Figure3A---SourceData---FLS2-flg22-CHX-BFA-FM4-64.tif]

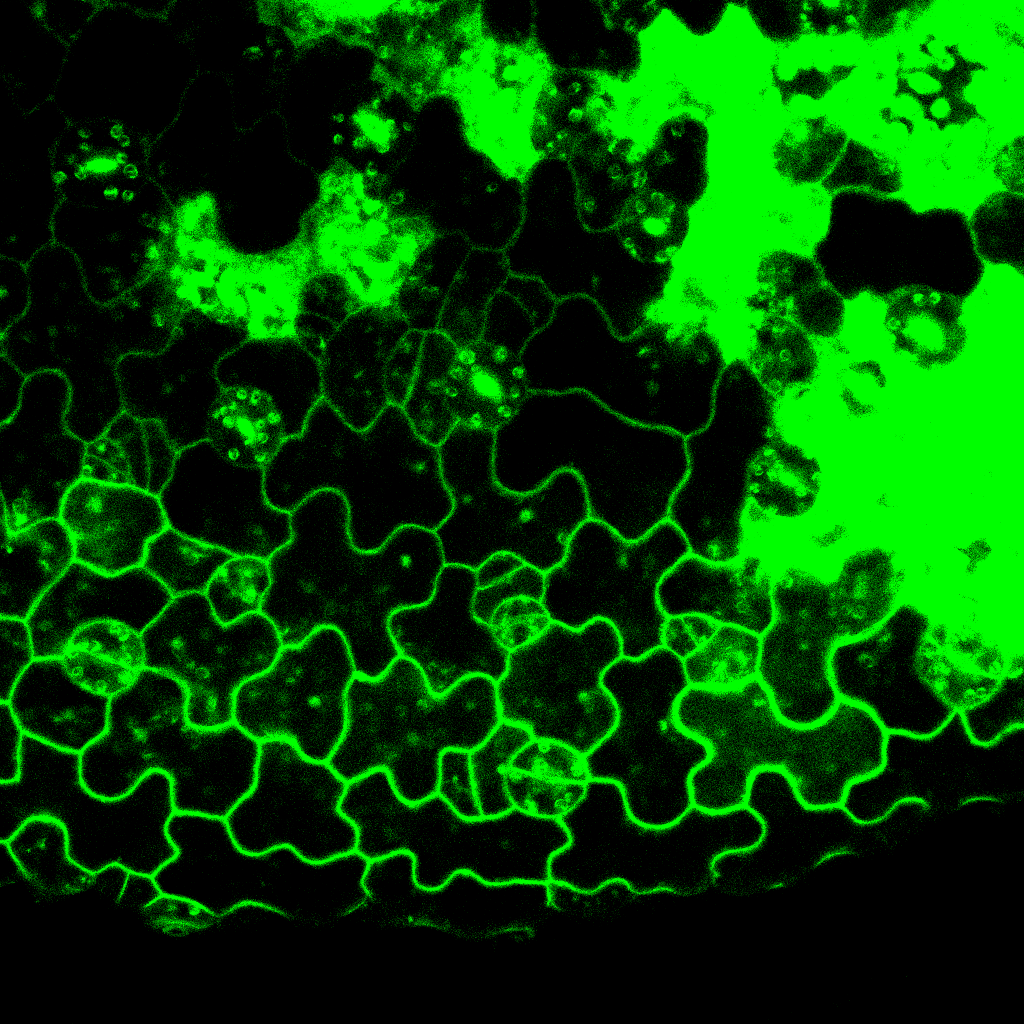

Supplement: Figure 3—source data 1. [file elife-91072-fig3-data1.zip › Figure 3—source data 1/Figure3A---SourceData---FLS2-flg22-CHX-BFA.tif]

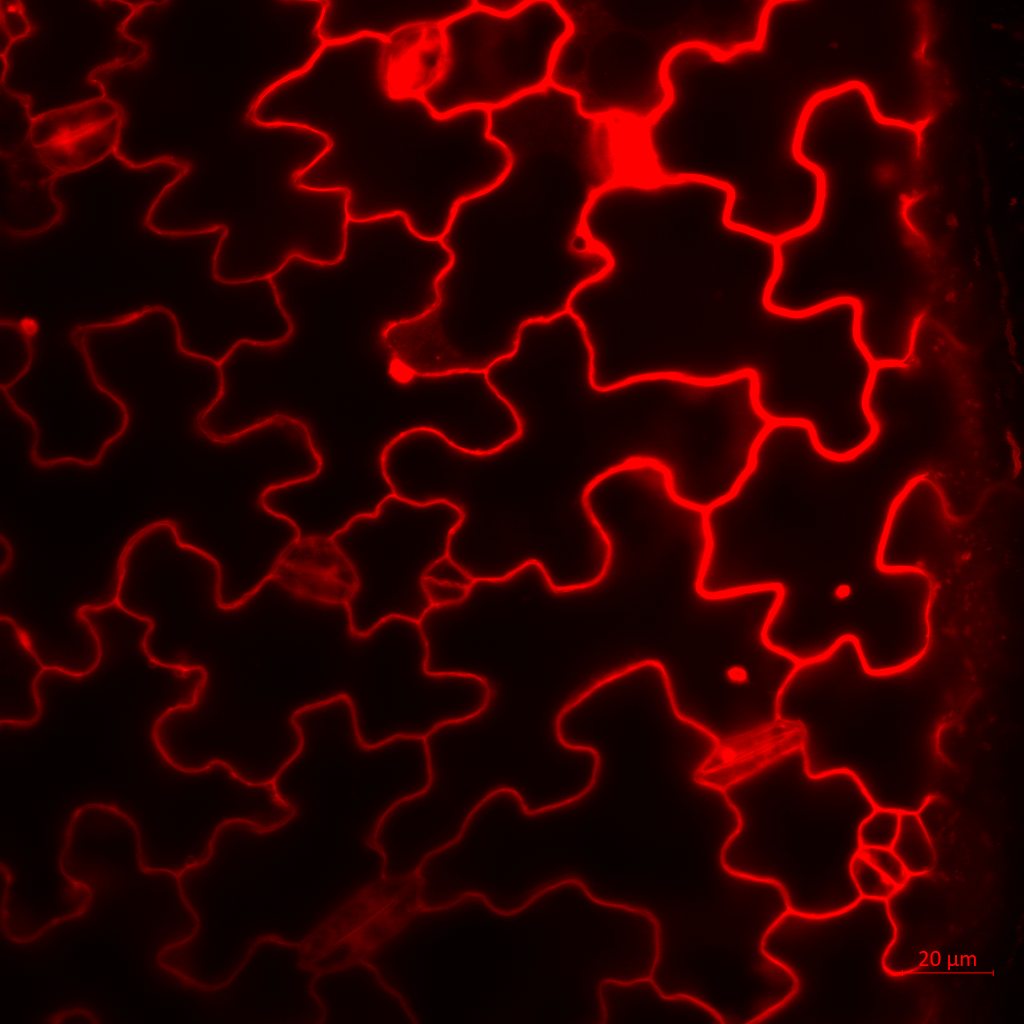

Supplement: Figure 3—source data 1. [file elife-91072-fig3-data1.zip › Figure 3—source data 1/Figure3A---SourceData---FLS2S938A-CHX-BFA-FM4-64.tif]

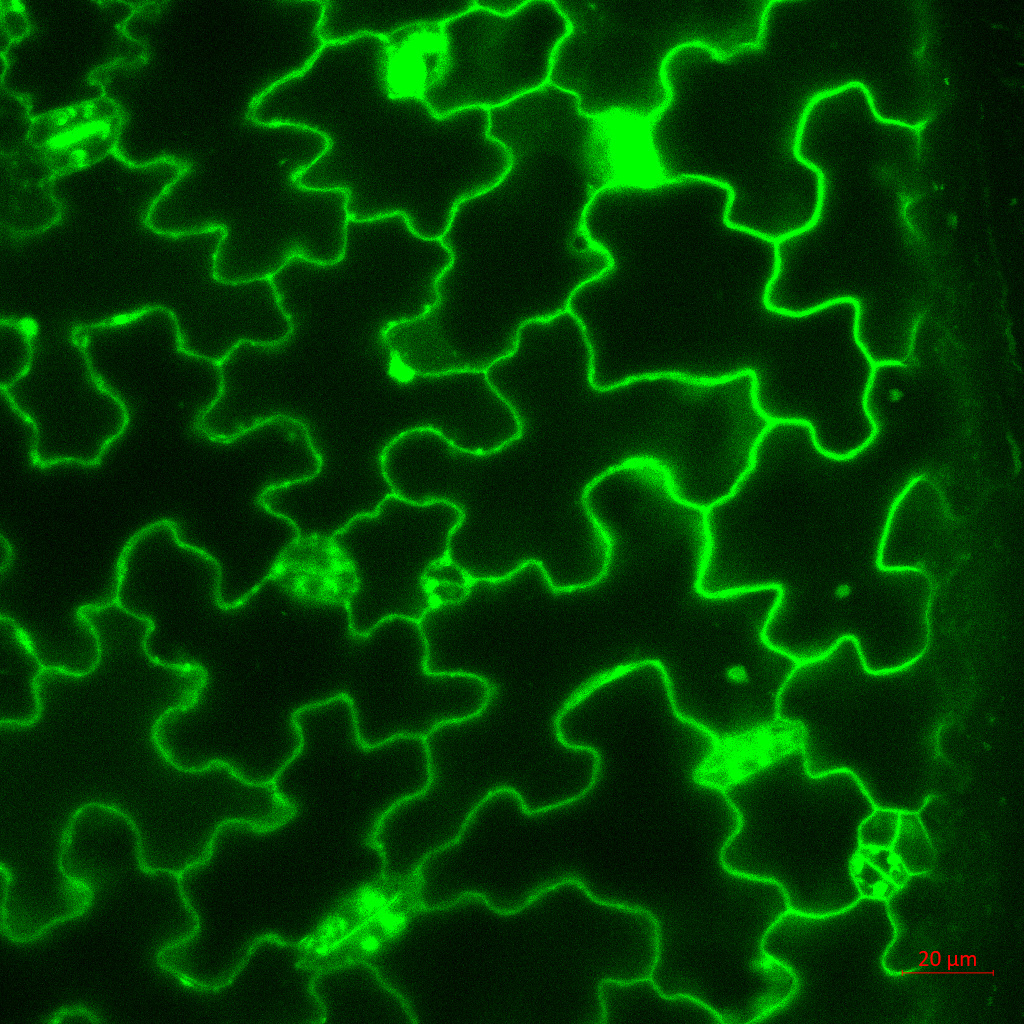

Supplement: Figure 3—source data 1. [file elife-91072-fig3-data1.zip › Figure 3—source data 1/Figure3A---SourceData---FLS2S938A-CHX-BFA.tif]

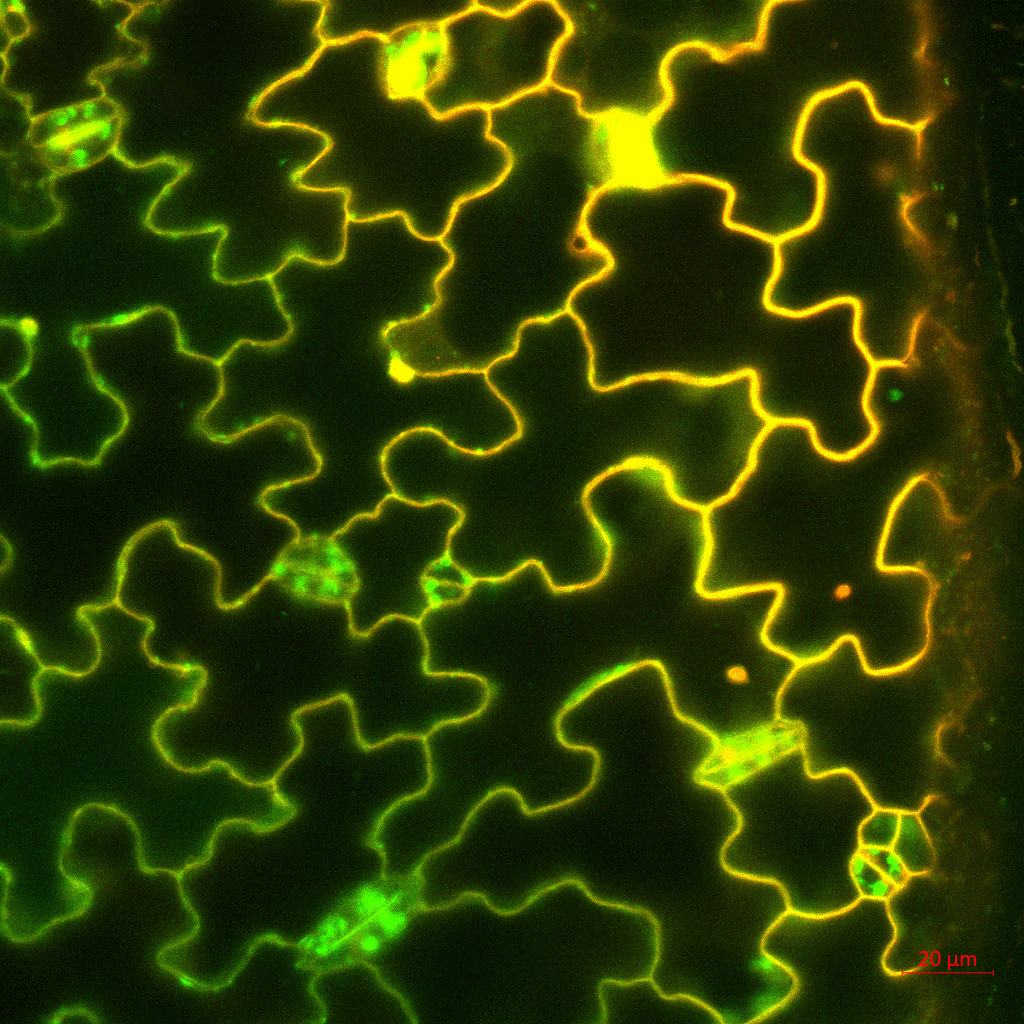

Supplement: Figure 3—source data 1. [file elife-91072-fig3-data1.zip › Figure 3—source data 1/Figure3A---SourceData---FLS2S938A-Merge .tif]

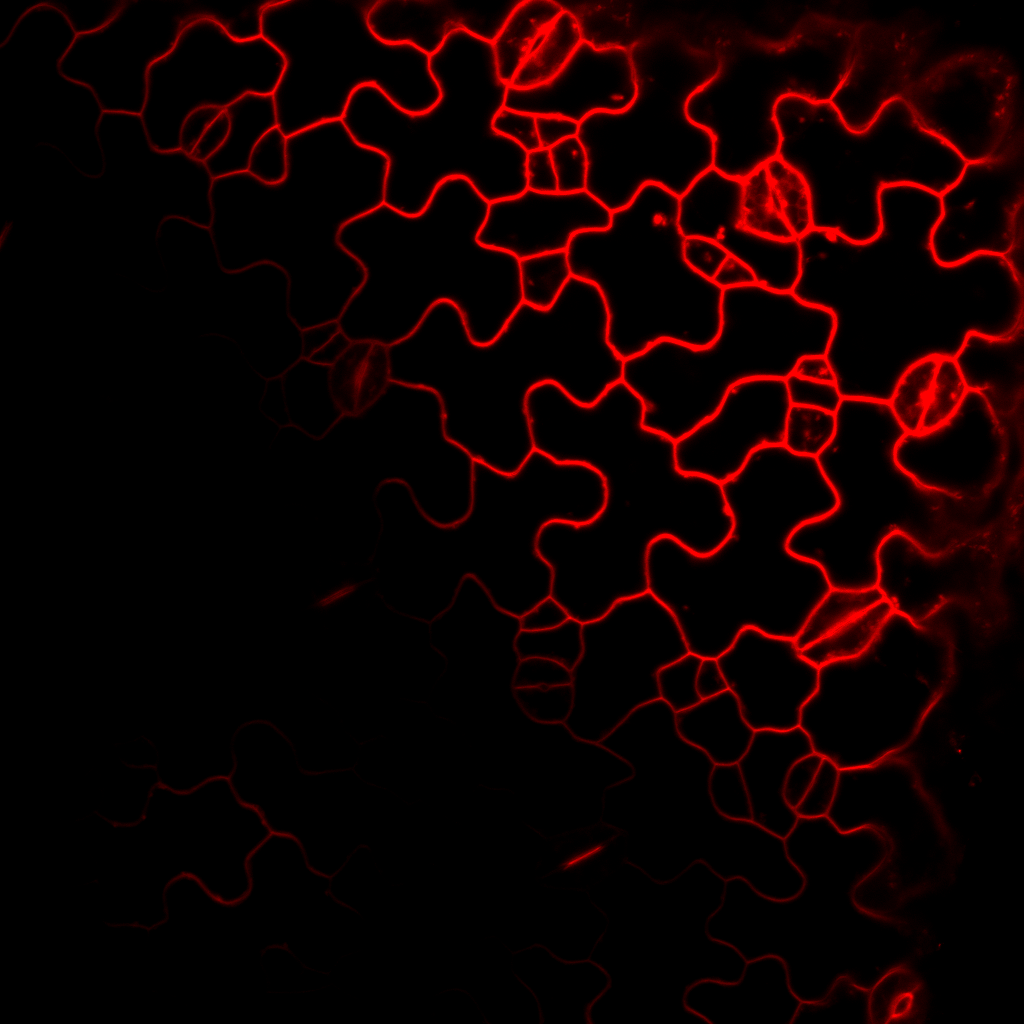

Supplement: Figure 3—source data 1. [file elife-91072-fig3-data1.zip › Figure 3—source data 1/Figure3A---SourceData---FLS2S938A-flg22-CHX-BFA-FM4-64.tif]

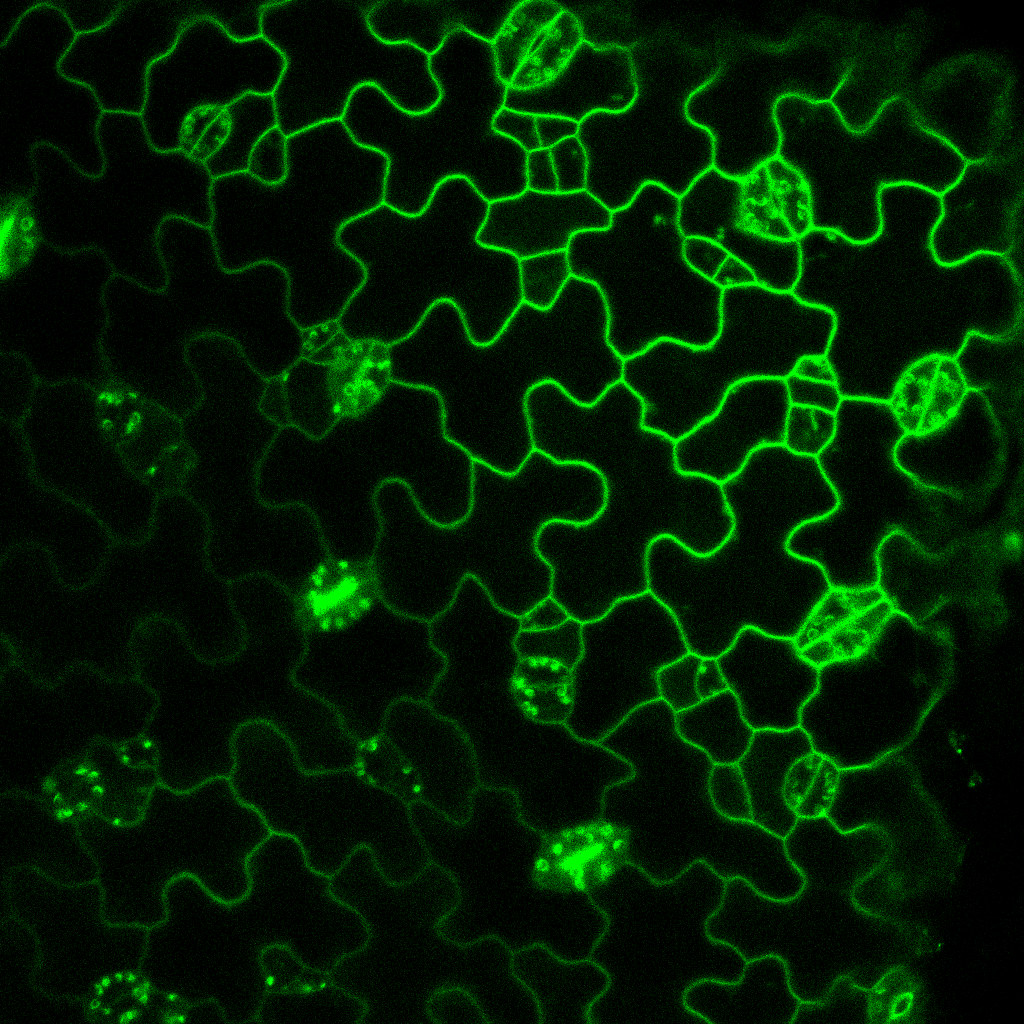

Supplement: Figure 3—source data 1. [file elife-91072-fig3-data1.zip › Figure 3—source data 1/Figure3A---SourceData---FLS2S938A-flg22-CHX-BFA.tif]

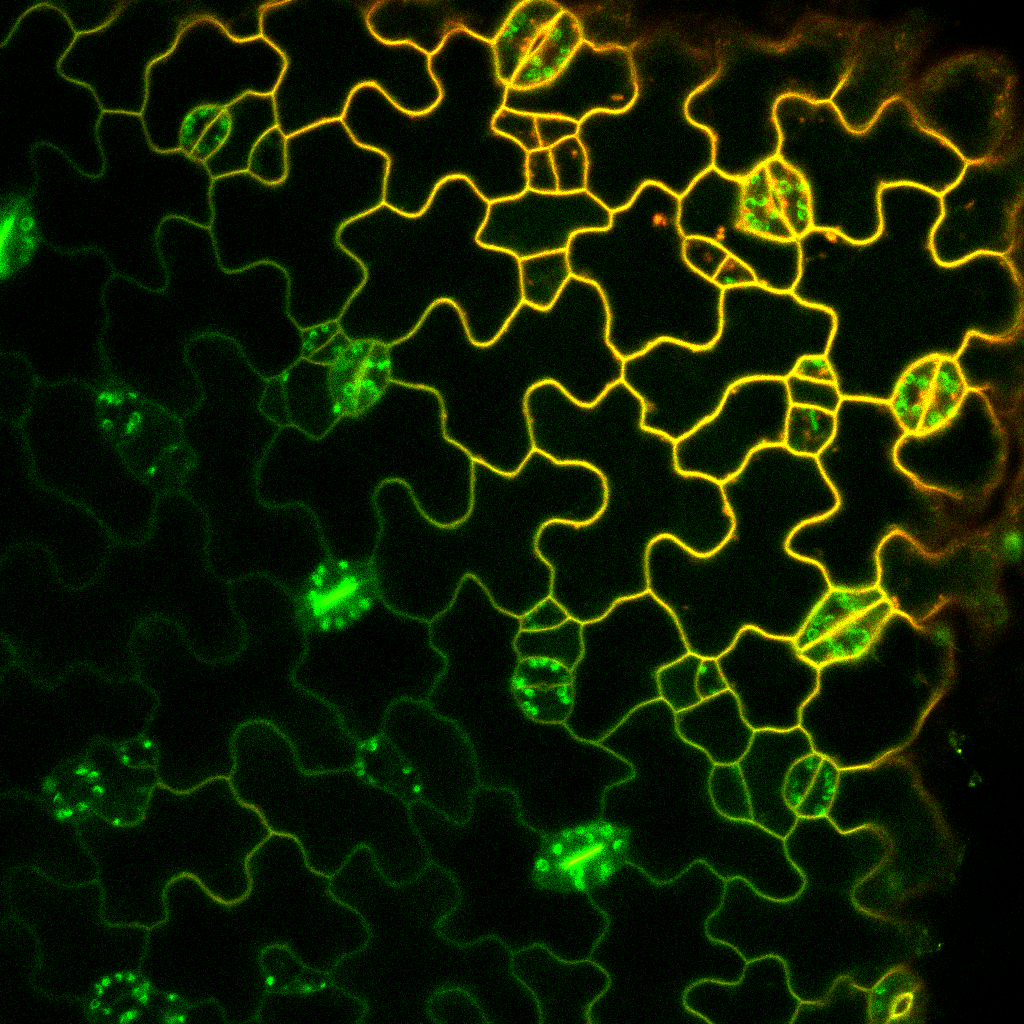

Supplement: Figure 3—source data 1. [file elife-91072-fig3-data1.zip › Figure 3—source data 1/Figure3A---SourceData---FLS2S938A-flg22-Merge .tif]

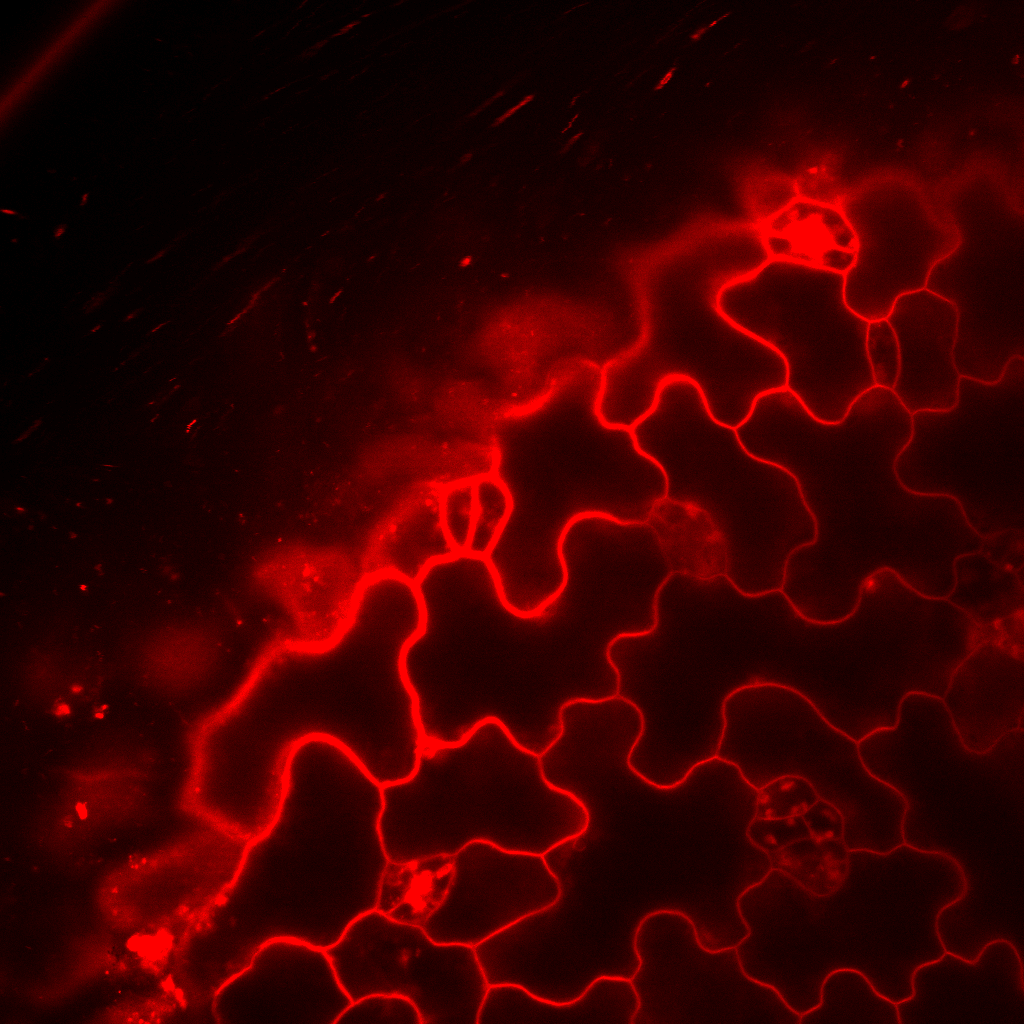

Supplement: Figure 3—source data 1. [file elife-91072-fig3-data1.zip › Figure 3—source data 1/Figure3A---SourceData---FLS2S938D-CHX-BFA-FM4-64.tif]

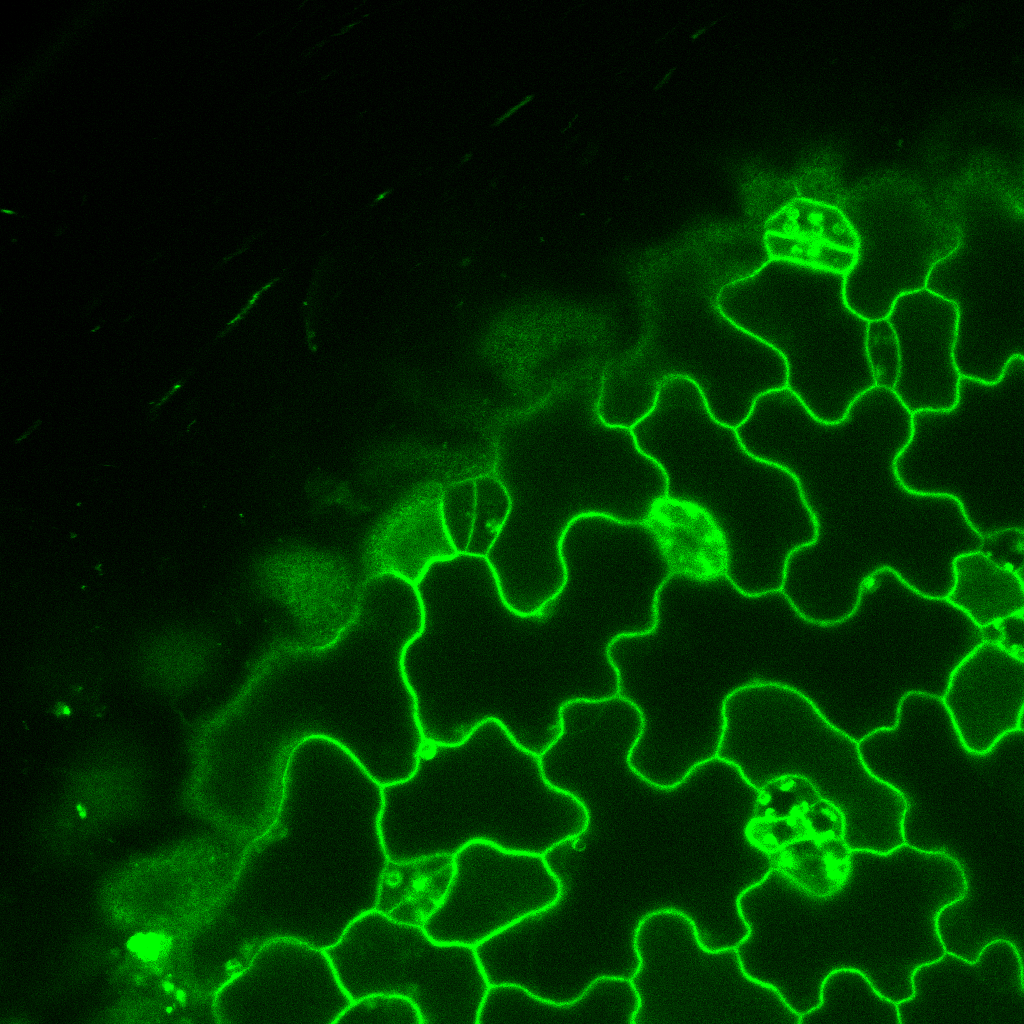

Supplement: Figure 3—source data 1. [file elife-91072-fig3-data1.zip › Figure 3—source data 1/Figure3A---SourceData---FLS2S938D-CHX-BFA.tif]

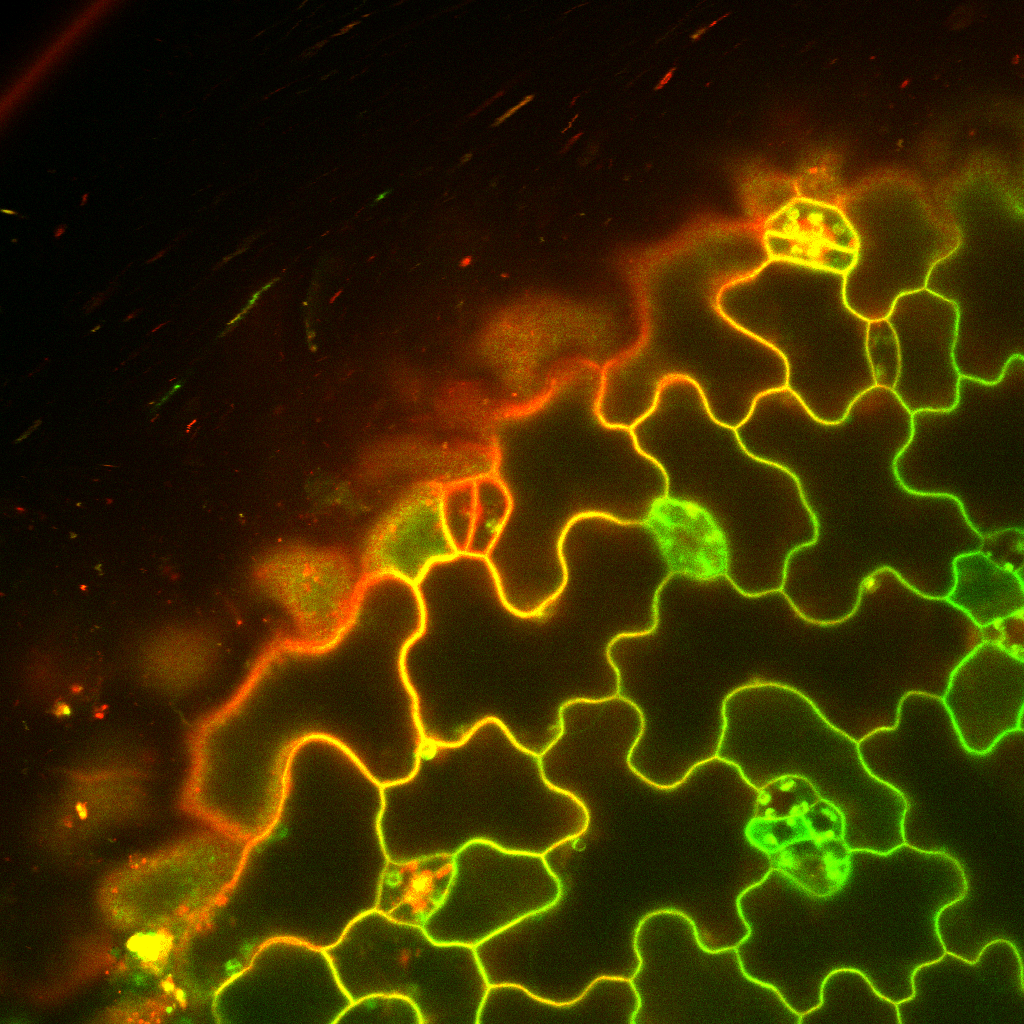

Supplement: Figure 3—source data 1. [file elife-91072-fig3-data1.zip › Figure 3—source data 1/Figure3A---SourceData---FLS2S938D-Merge .tif]

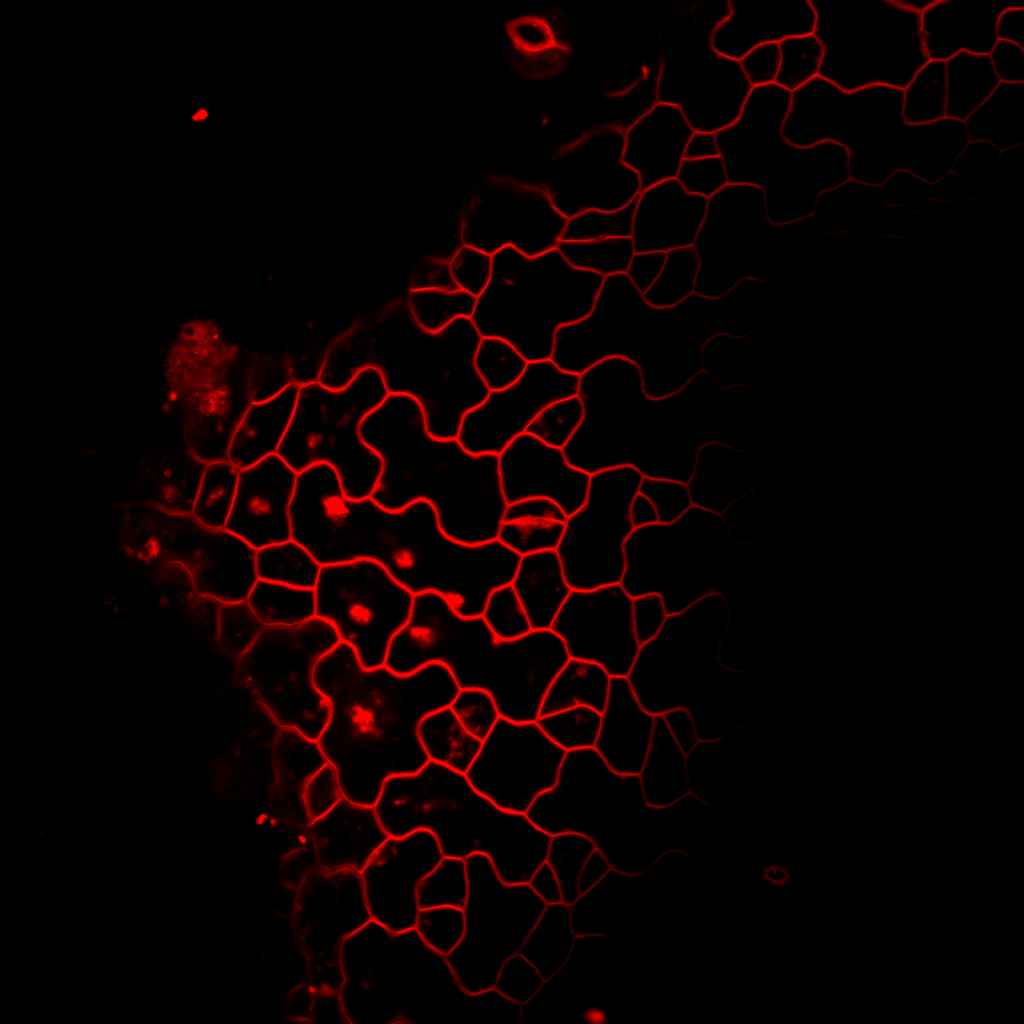

Supplement: Figure 3—source data 1. [file elife-91072-fig3-data1.zip › Figure 3—source data 1/Figure3A---SourceData---FLS2S938D-flg22-CHX-BFA-FM4-64.tif]

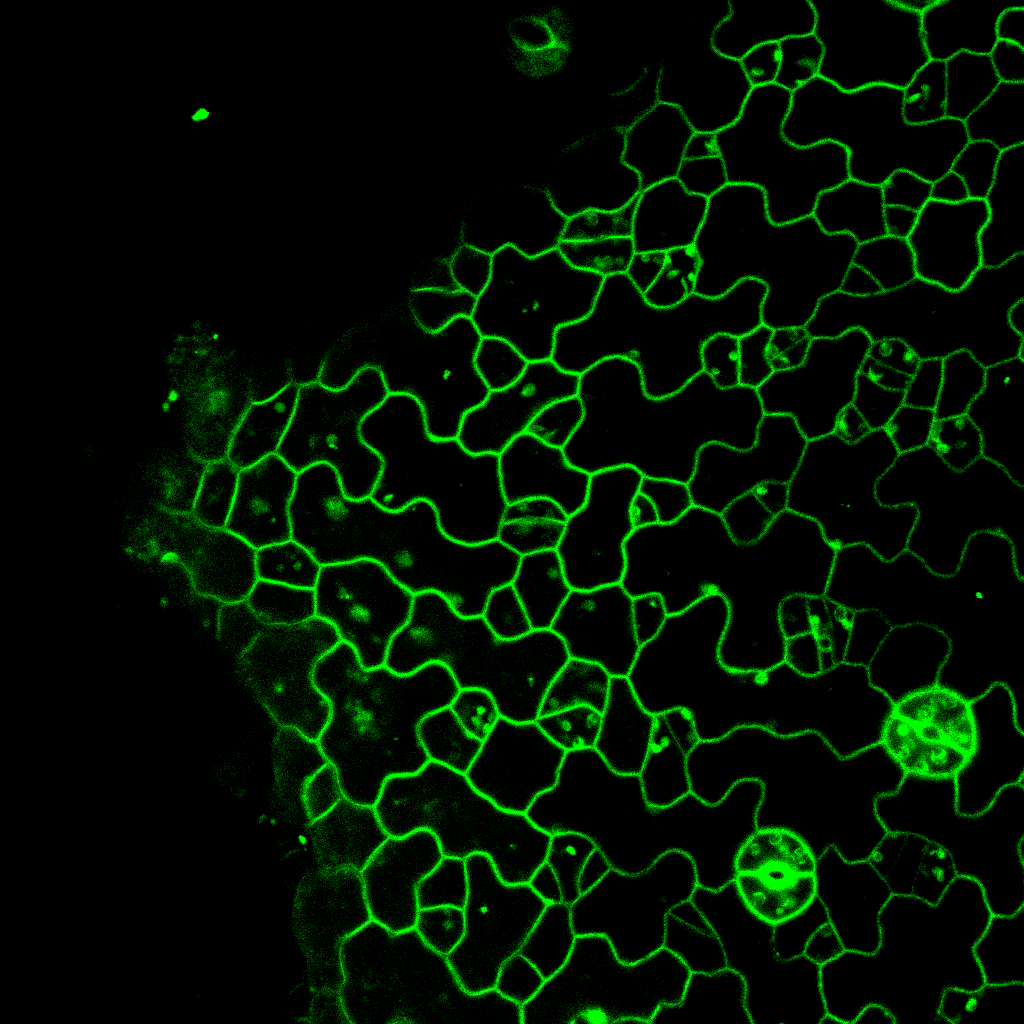

Supplement: Figure 3—source data 1. [file elife-91072-fig3-data1.zip › Figure 3—source data 1/Figure3A---SourceData---FLS2S938D-flg22-CHX-BFA.tif]

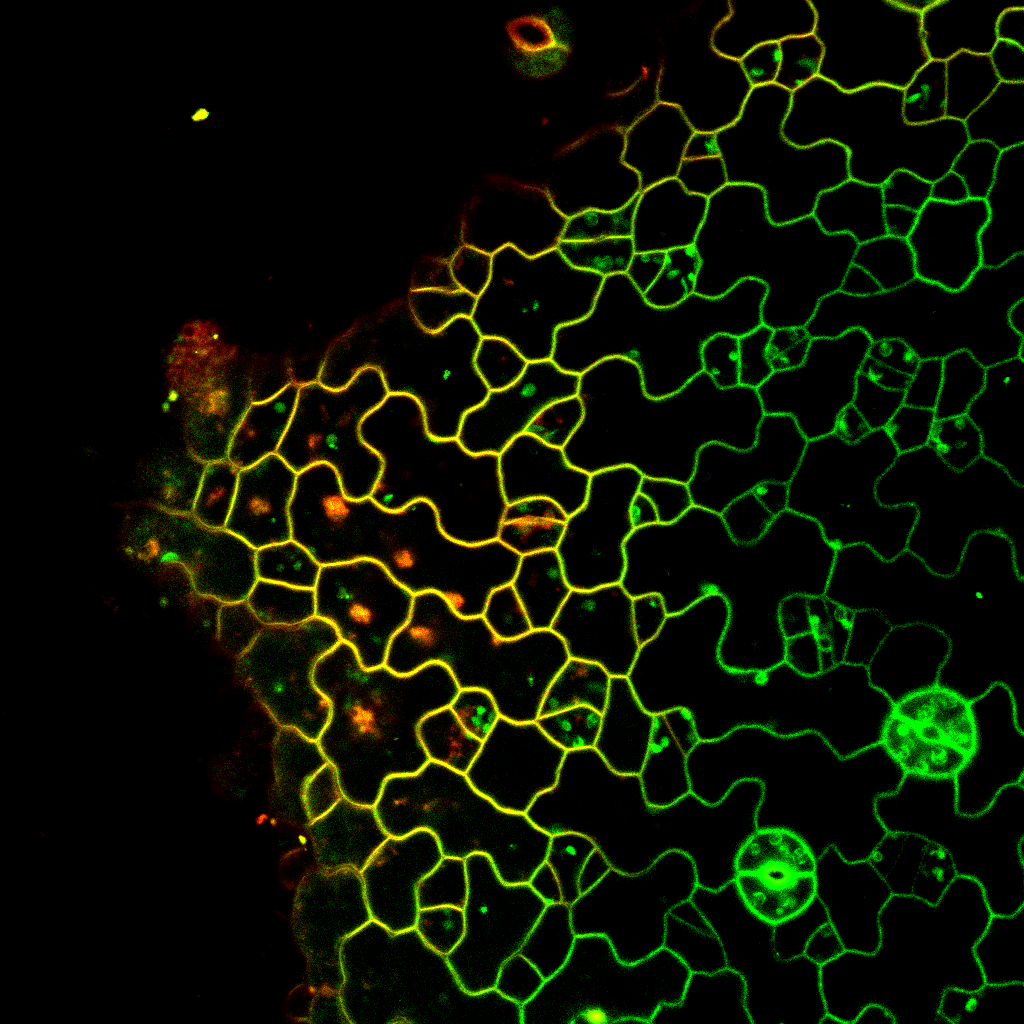

Supplement: Figure 3—source data 1. [file elife-91072-fig3-data1.zip › Figure 3—source data 1/Figure3A---SourceData---FLS2S938D-flg22-Merge.tif]

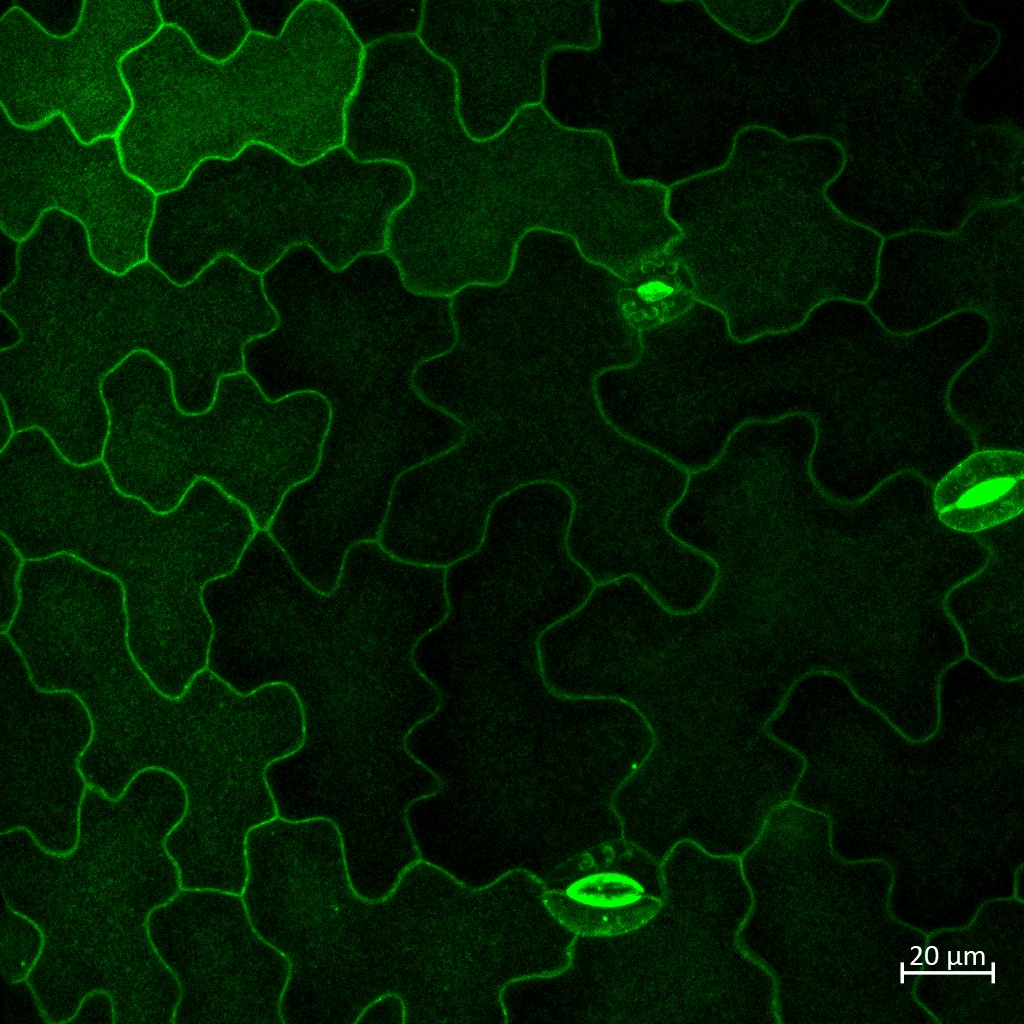

Supplement: Figure 3—source data 2. [file elife-91072-fig3-data2.zip › Figure 3—source data 2/Figure3B---SourceData---FLS2-0min.jpg]

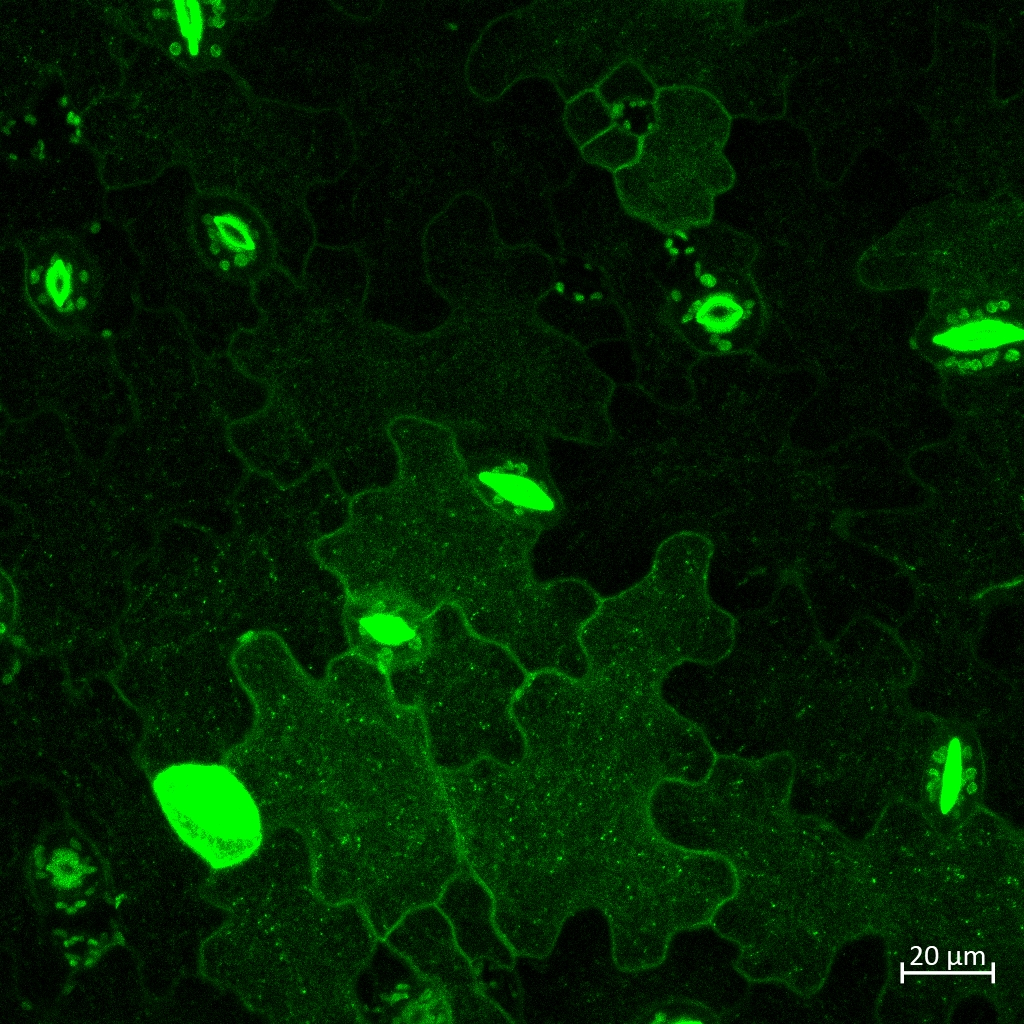

Supplement: Figure 3—source data 2. [file elife-91072-fig3-data2.zip › Figure 3—source data 2/Figure3B---SourceData---FLS2-flg22-15min.jpg]

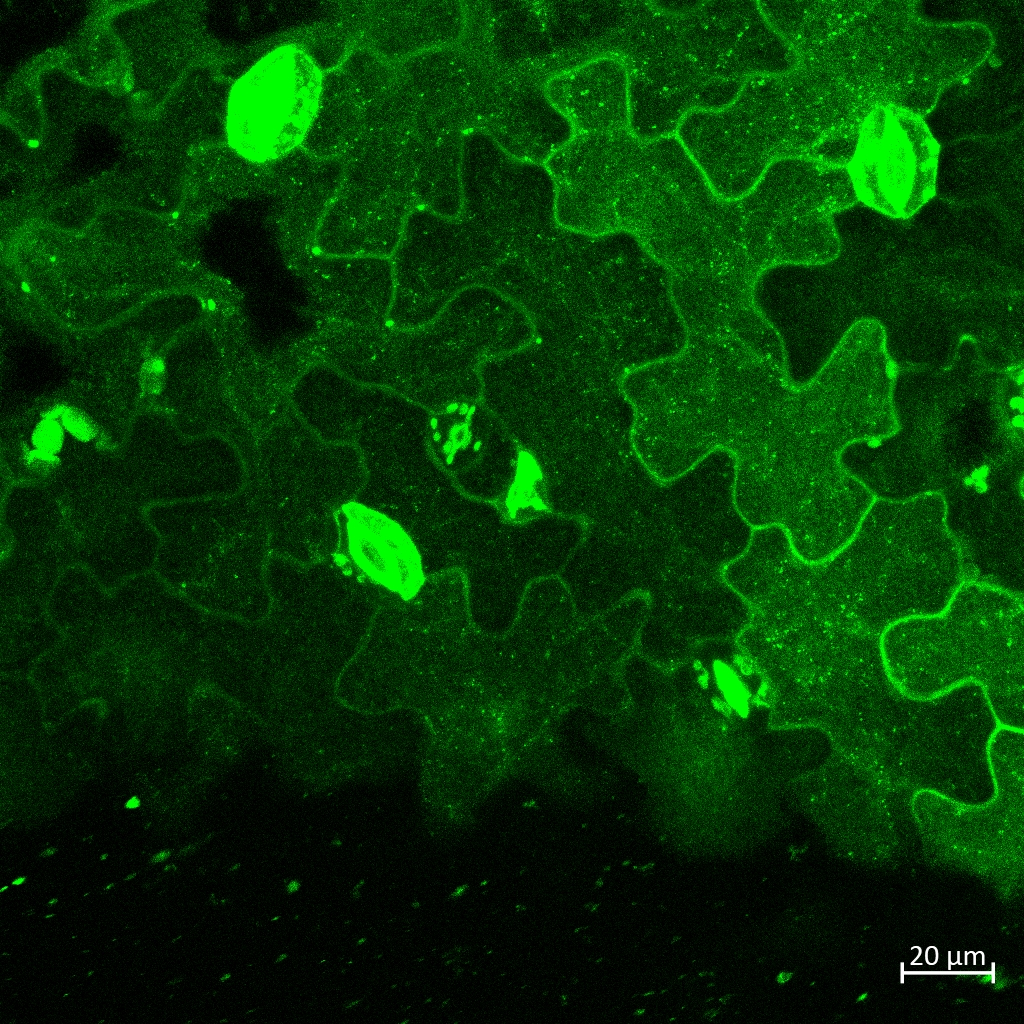

Supplement: Figure 3—source data 2. [file elife-91072-fig3-data2.zip › Figure 3—source data 2/Figure3B---SourceData---FLS2-flg22-30min.jpg]

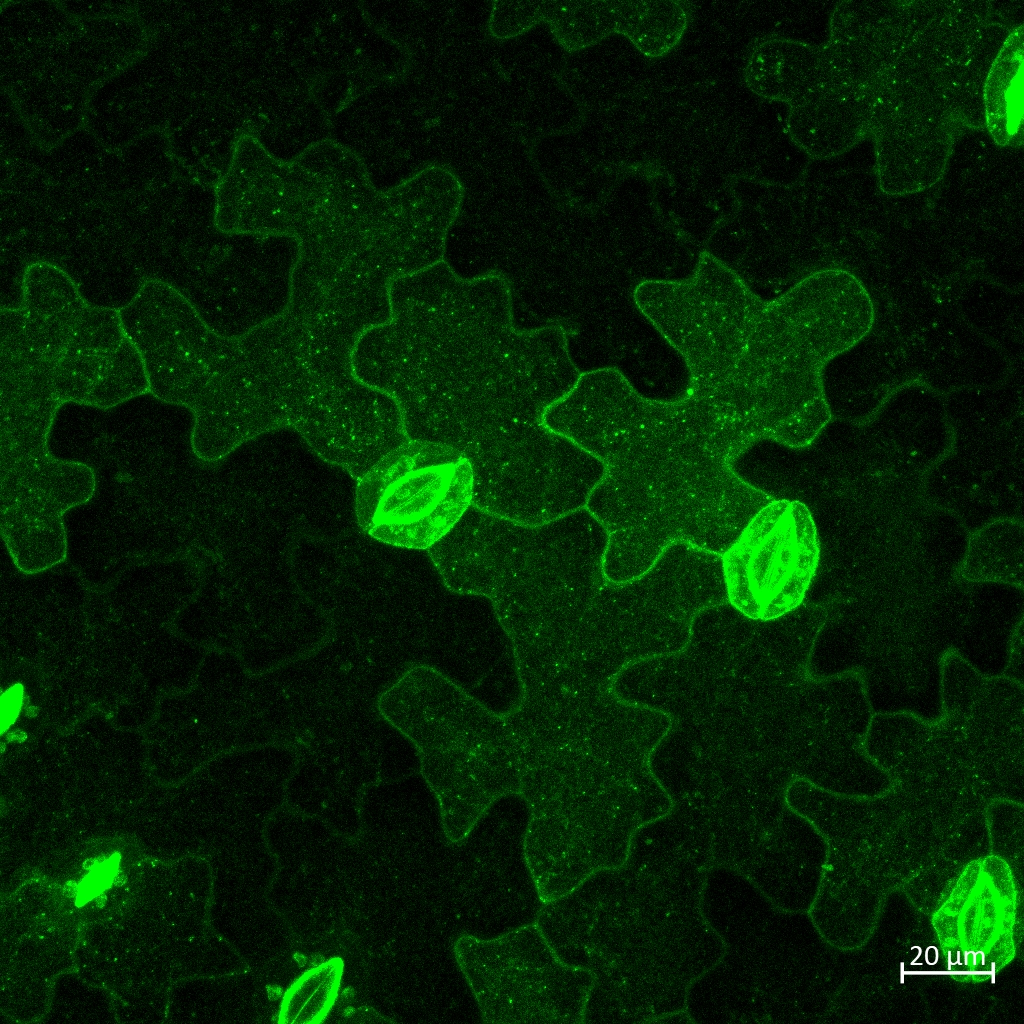

Supplement: Figure 3—source data 2. [file elife-91072-fig3-data2.zip › Figure 3—source data 2/Figure3B---SourceData---FLS2-flg22-60min.jpg]

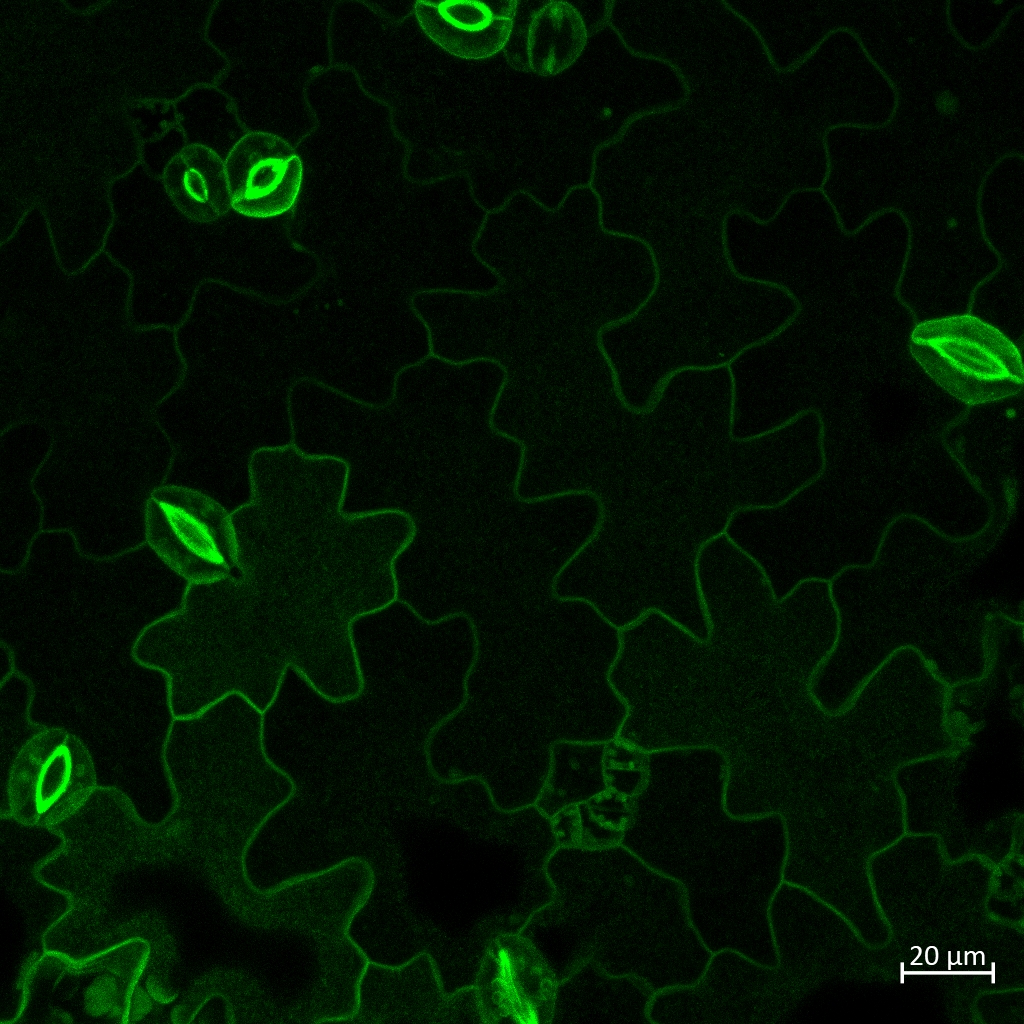

Supplement: Figure 3—source data 2. [file elife-91072-fig3-data2.zip › Figure 3—source data 2/Figure3B---SourceData---FLS2S938A-0min.jpg]

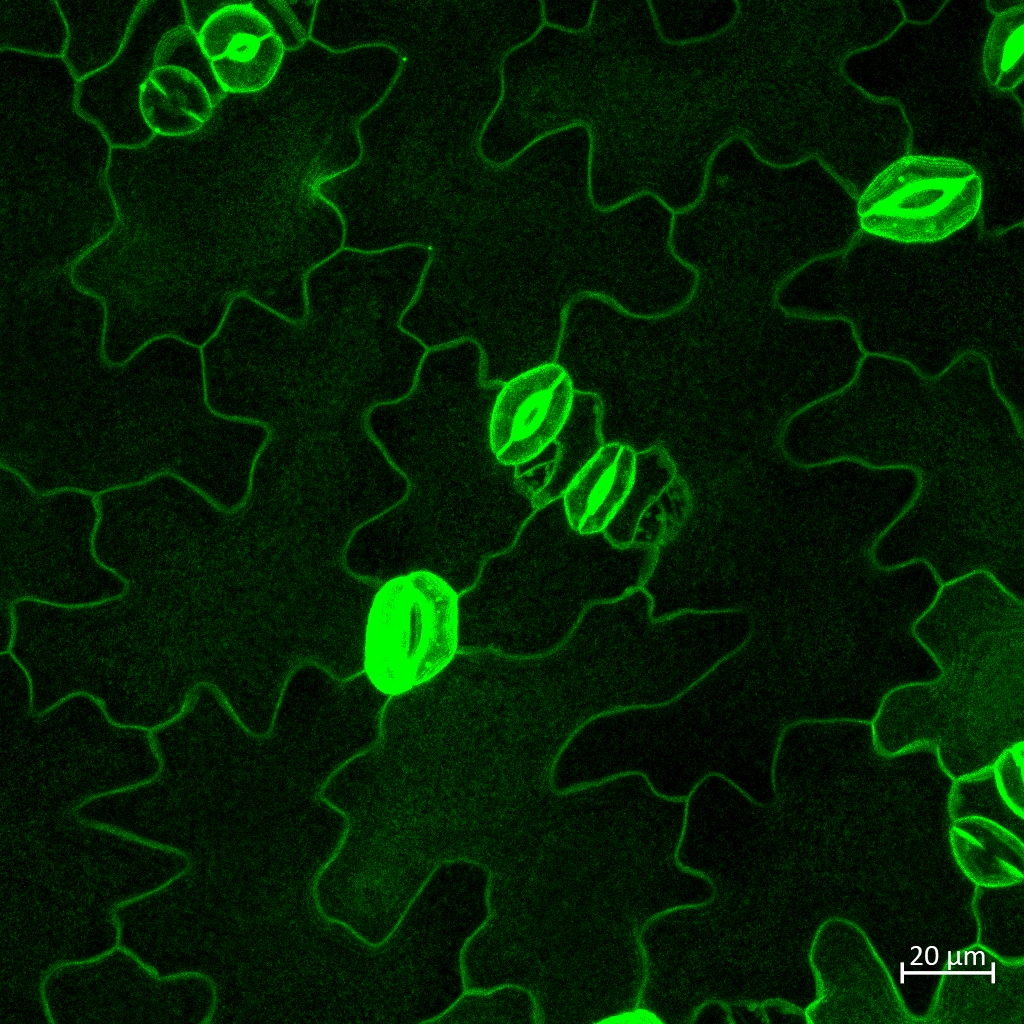

Supplement: Figure 3—source data 2. [file elife-91072-fig3-data2.zip › Figure 3—source data 2/Figure3B---SourceData---FLS2S938A-flg22-15min.jpg]

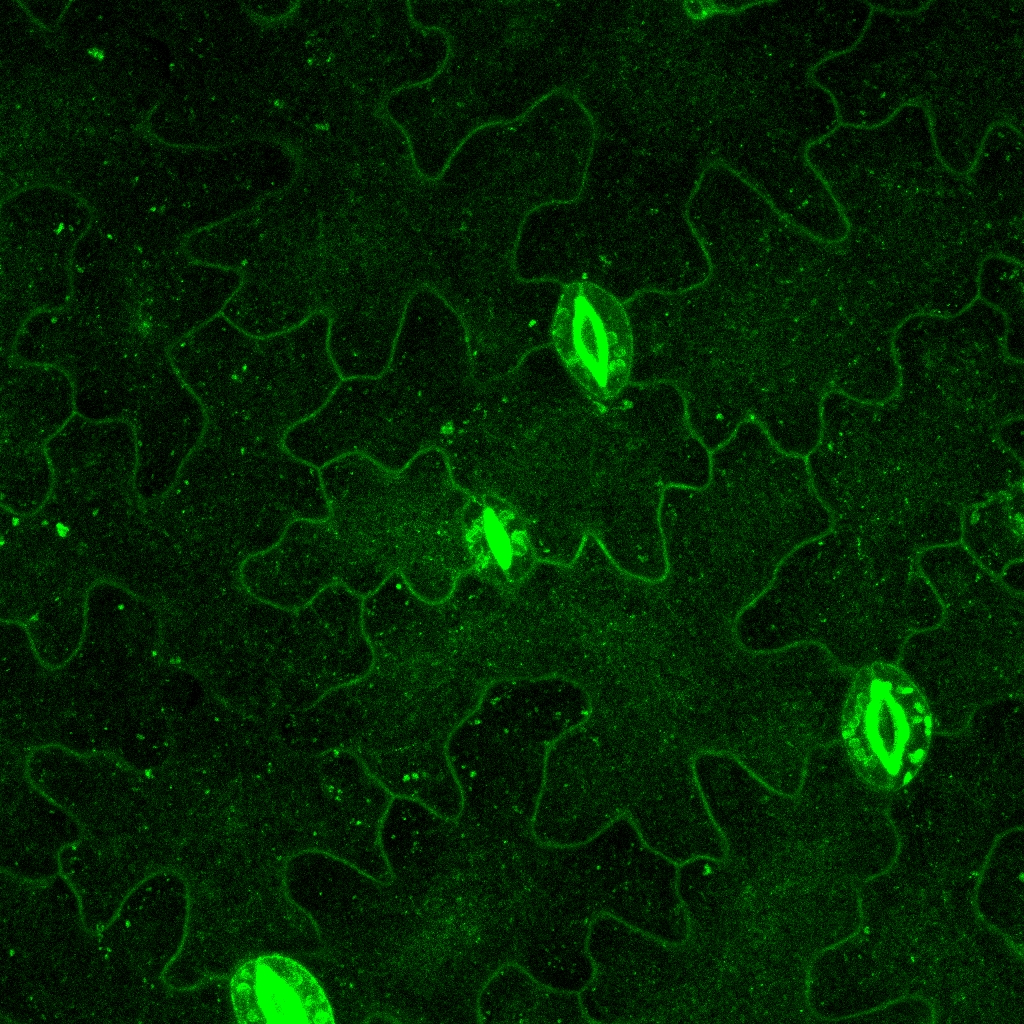

Supplement: Figure 3—source data 2. [file elife-91072-fig3-data2.zip › Figure 3—source data 2/Figure3B---SourceData---FLS2S938A-flg22-30min.jpg]

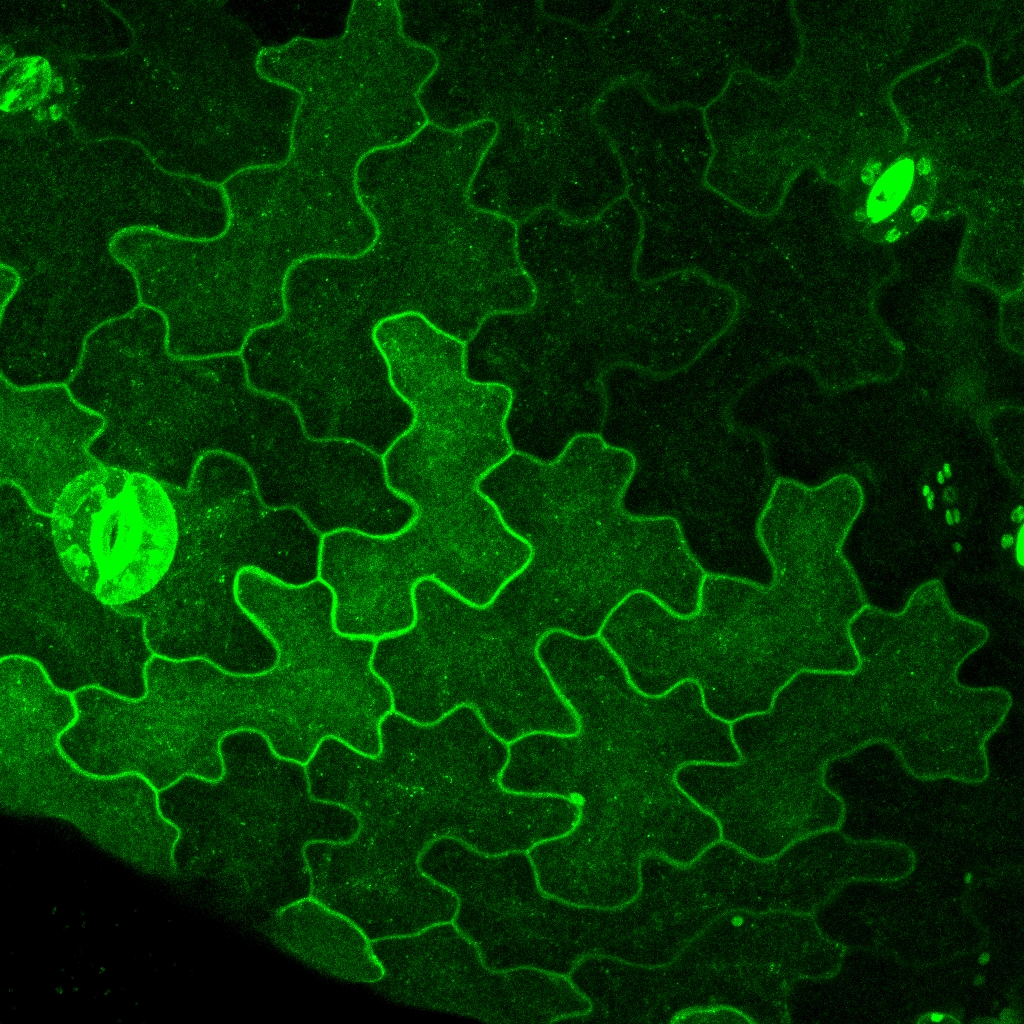

Supplement: Figure 3—source data 2. [file elife-91072-fig3-data2.zip › Figure 3—source data 2/Figure3B---SourceData---FLS2S938A-flg22-60min.jpg]

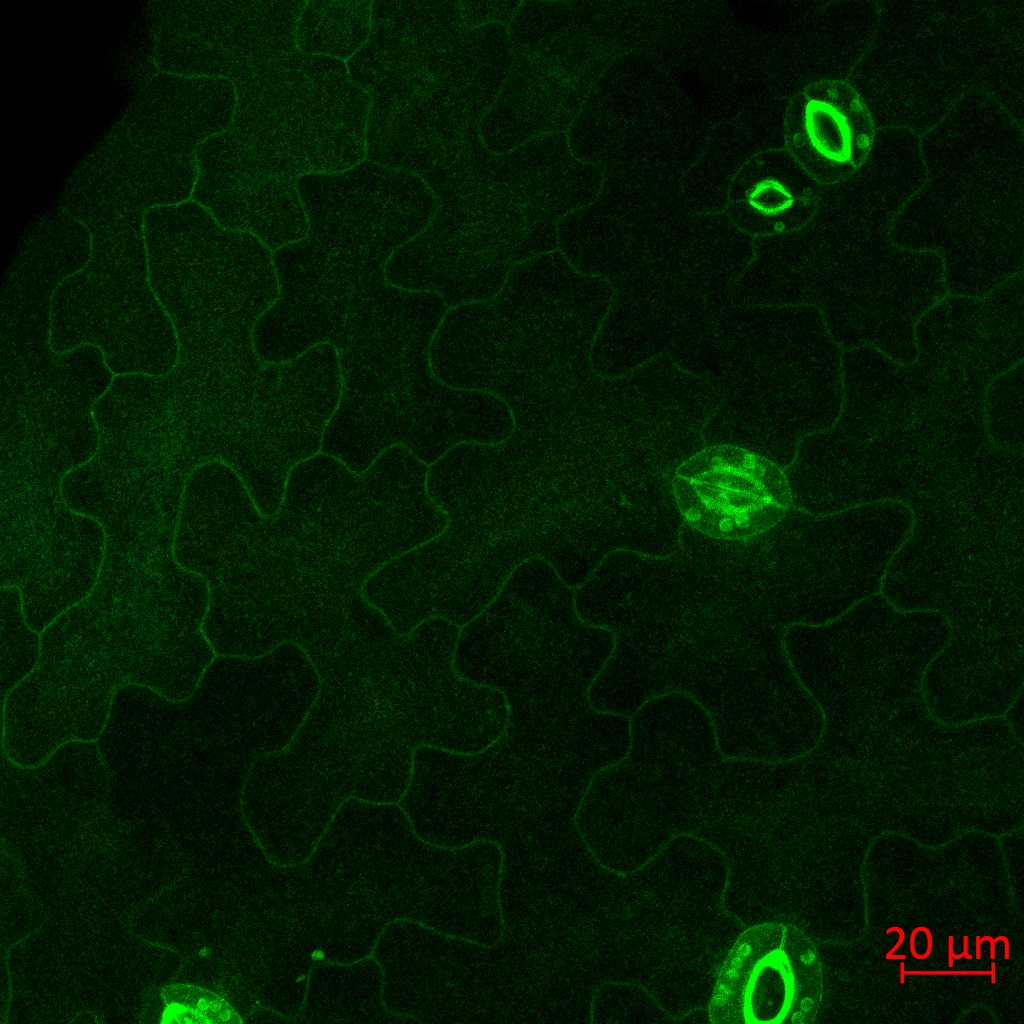

Supplement: Figure 3—source data 2. [file elife-91072-fig3-data2.zip › Figure 3—source data 2/Figure3B---SourceData---FLS2S938D-0min.jpg]

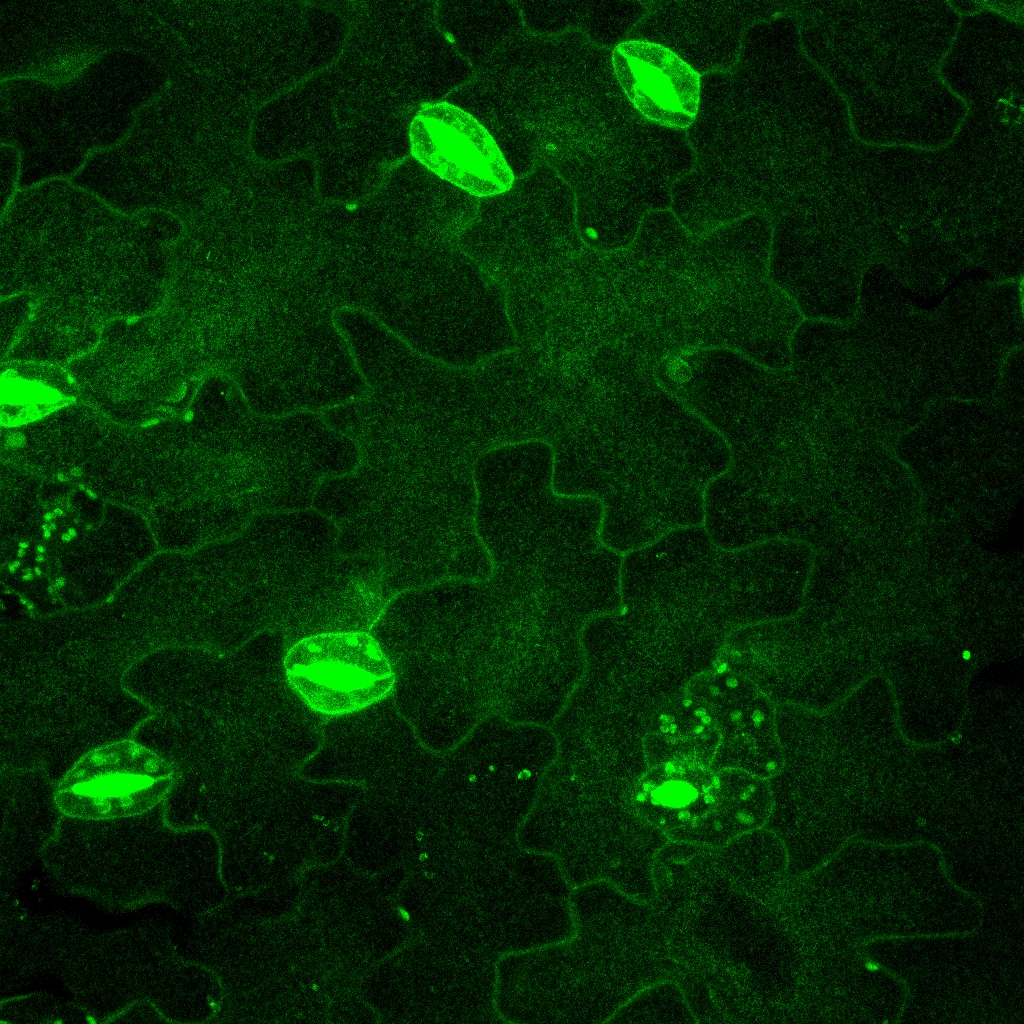

Supplement: Figure 3—source data 2. [file elife-91072-fig3-data2.zip › Figure 3—source data 2/Figure3B---SourceData---FLS2S938D-flg22-15min.jpg]

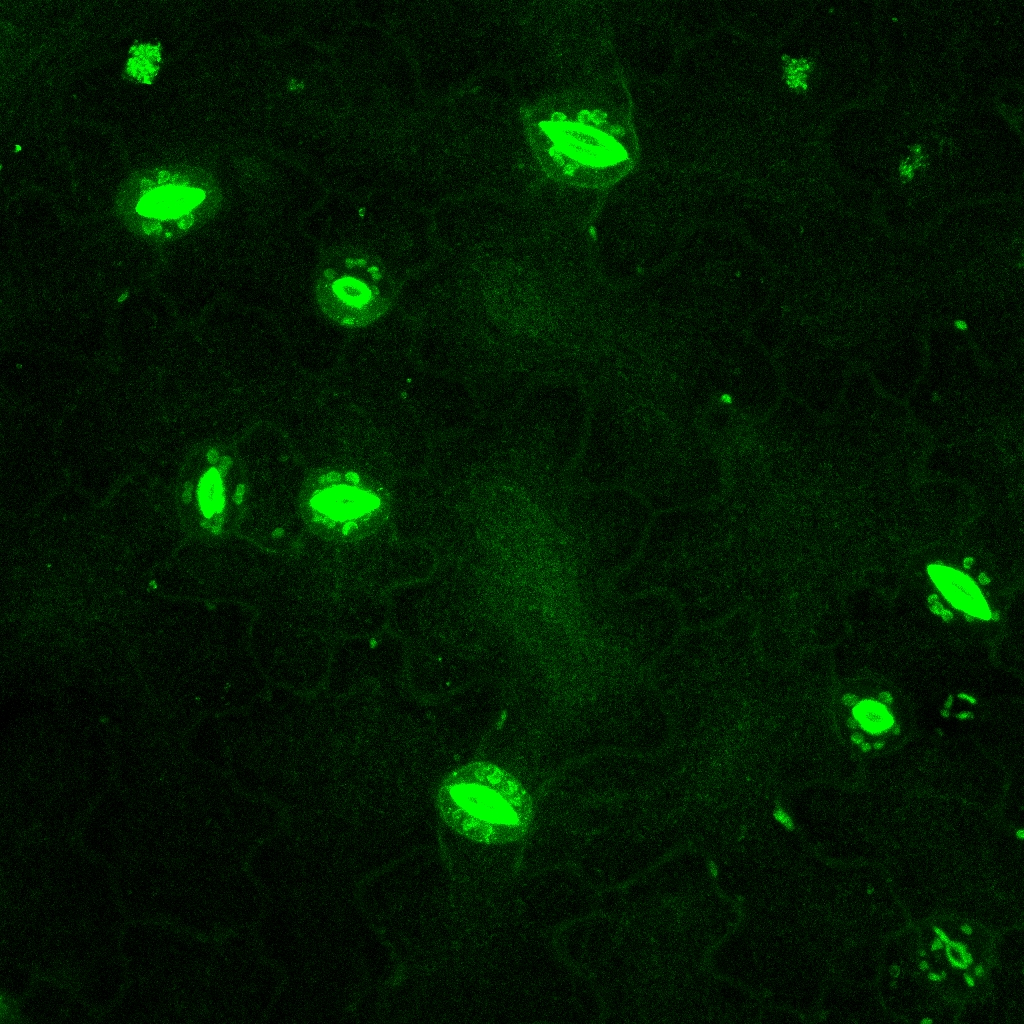

Supplement: Figure 3—source data 2. [file elife-91072-fig3-data2.zip › Figure 3—source data 2/Figure3B---SourceData---FLS2S938D-flg22-30min.jpg]

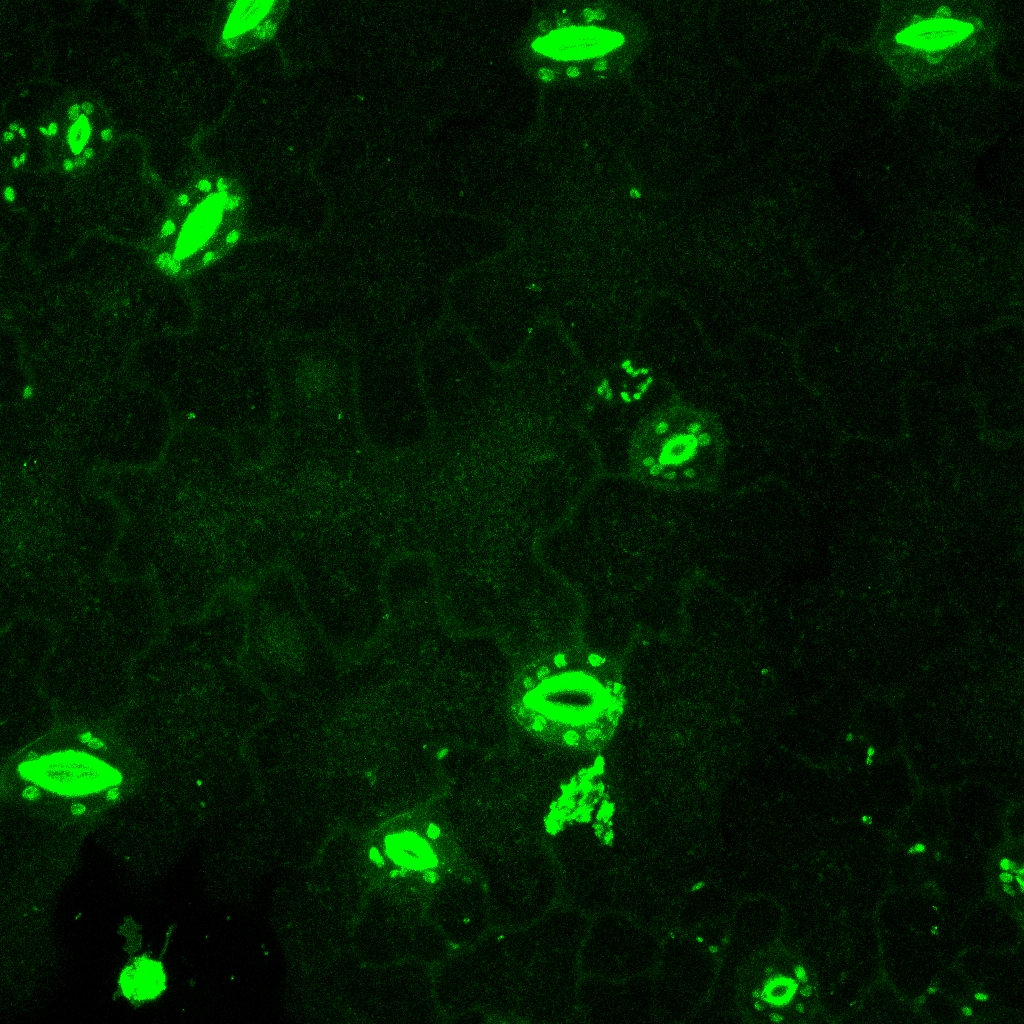

Supplement: Figure 3—source data 2. [file elife-91072-fig3-data2.zip › Figure 3—source data 2/Figure3B---SourceData---FLS2S938D-flg22-60min.jpg]

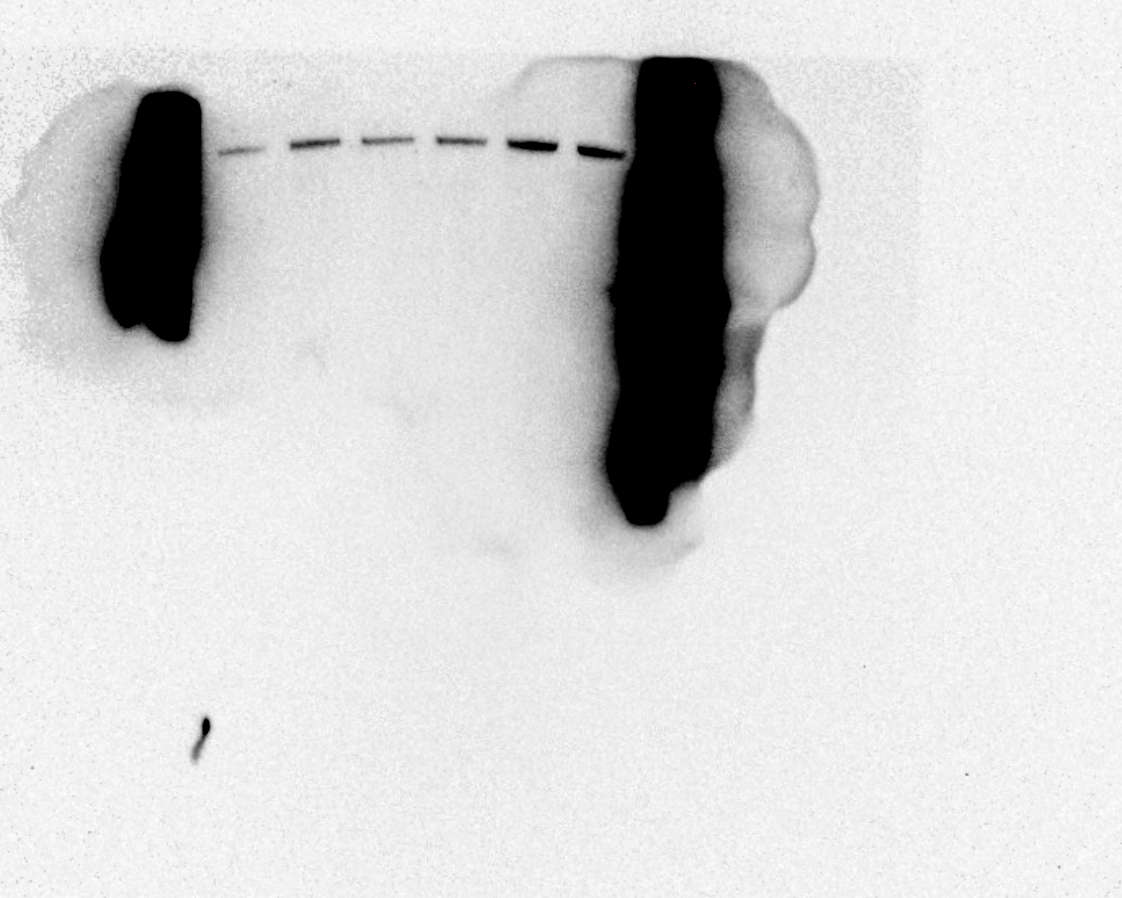

Supplement: Figure 3—source data 5. [file elife-91072-fig3-data5.zip › Figure 3—source data 5/Figure3E---SourceData1.jpg]

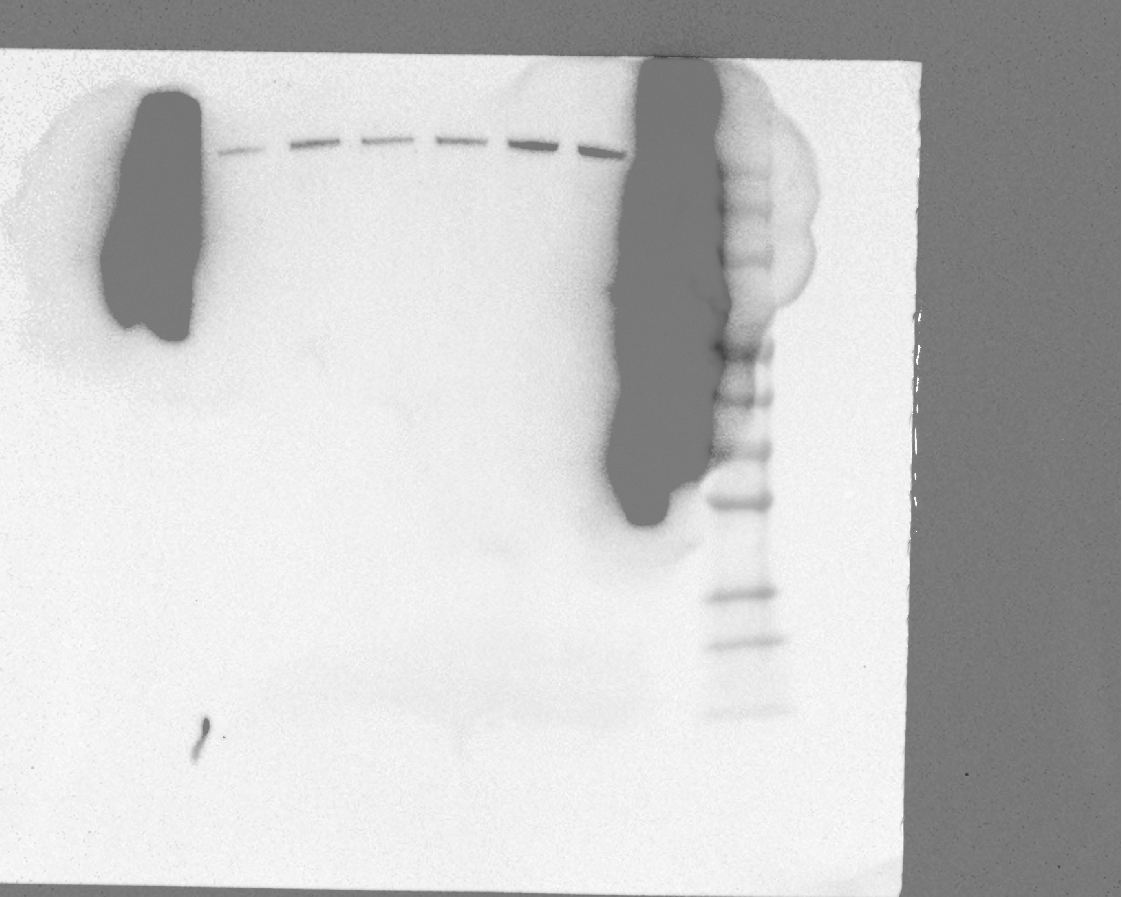

Supplement: Figure 3—source data 5. [file elife-91072-fig3-data5.zip › Figure 3—source data 5/Figure3E---SourceData2.jpg]

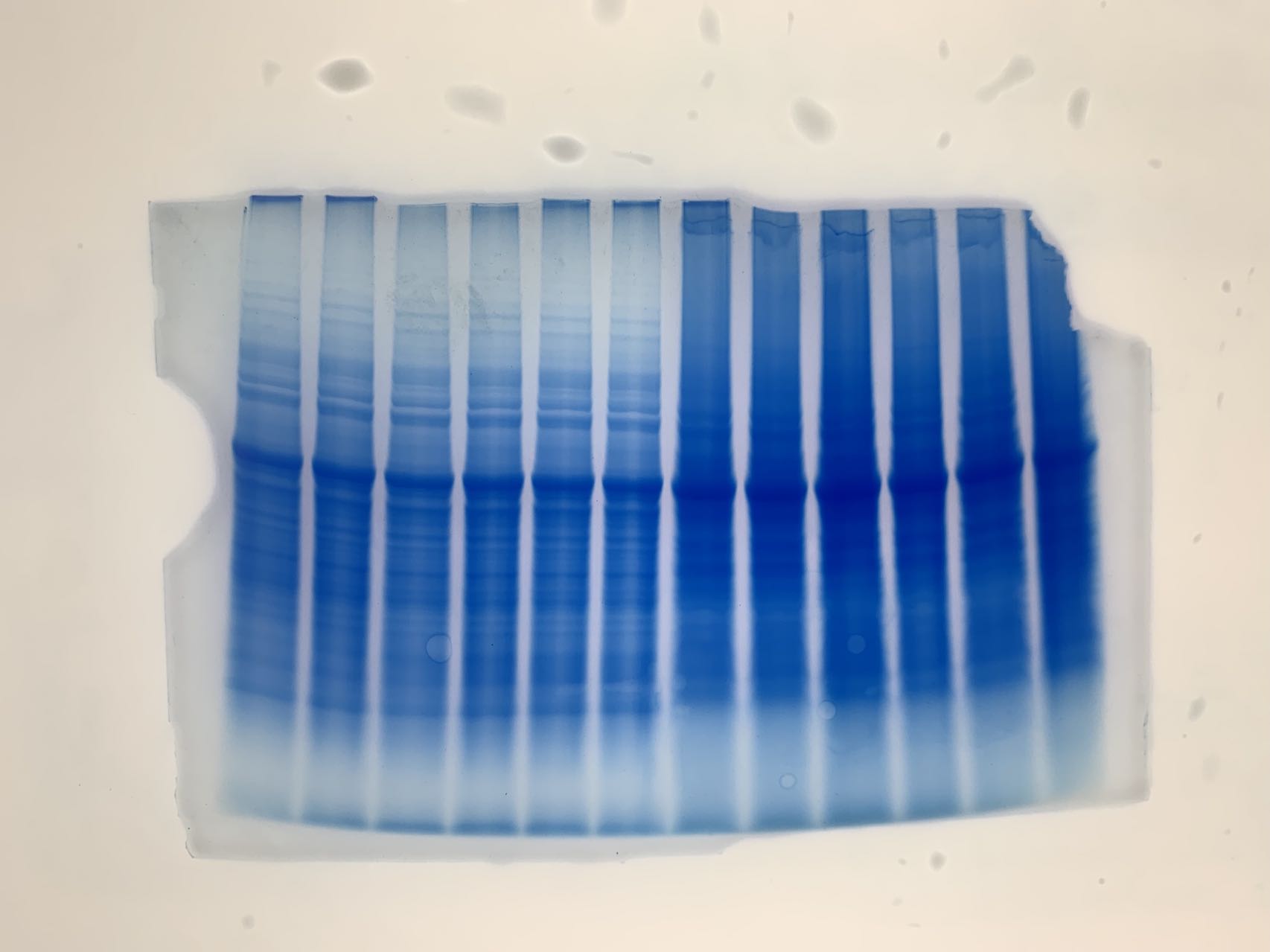

Supplement: Figure 3—source data 5. [file elife-91072-fig3-data5.zip › Figure 3—source data 5/Figure3E---SourceData3.jpg]

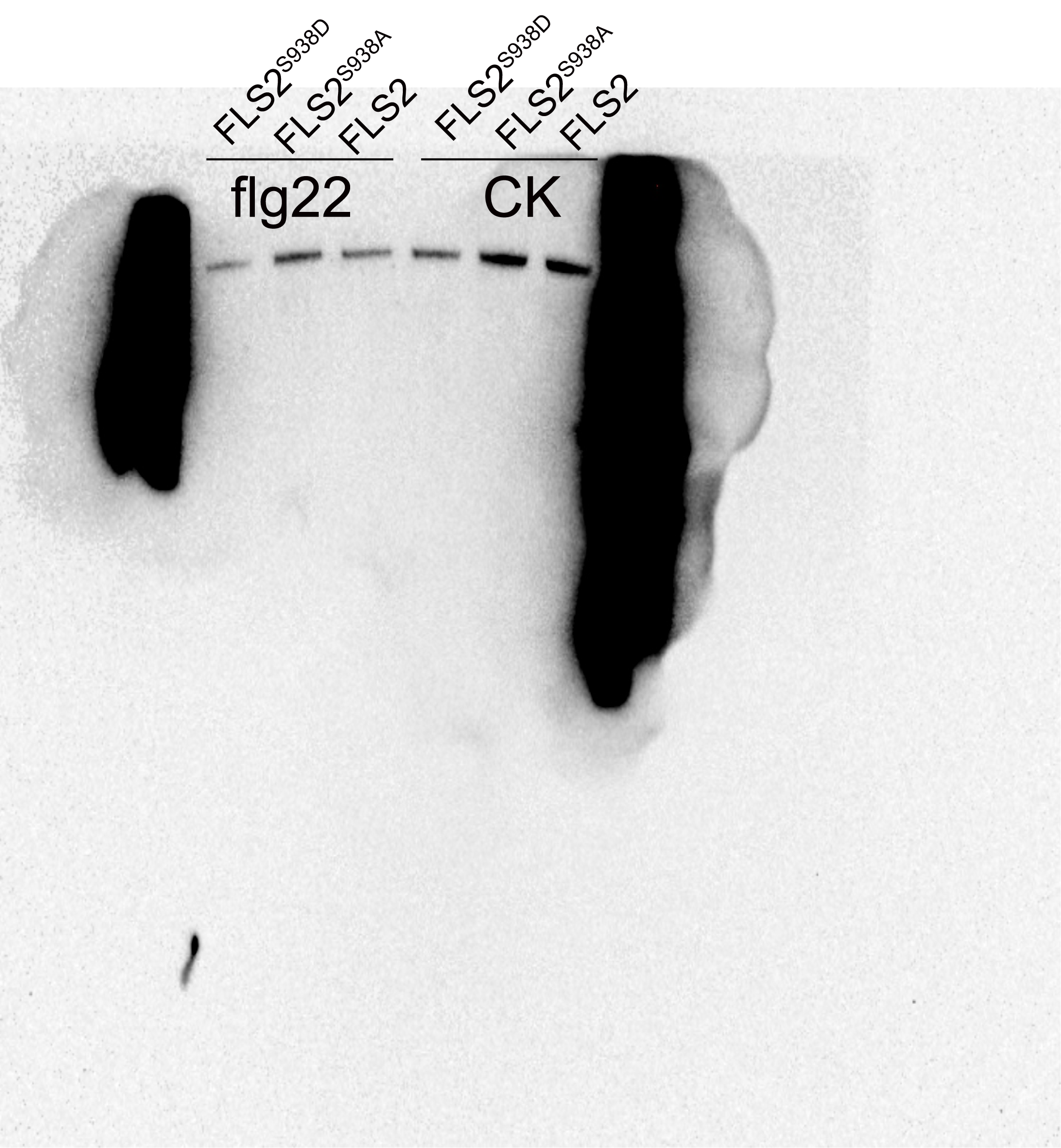

Supplement: Figure 3—source data 6. [file elife-91072-fig3-data6.zip › Figure 3—source data 6/Figure3E---SourceData1.tif]

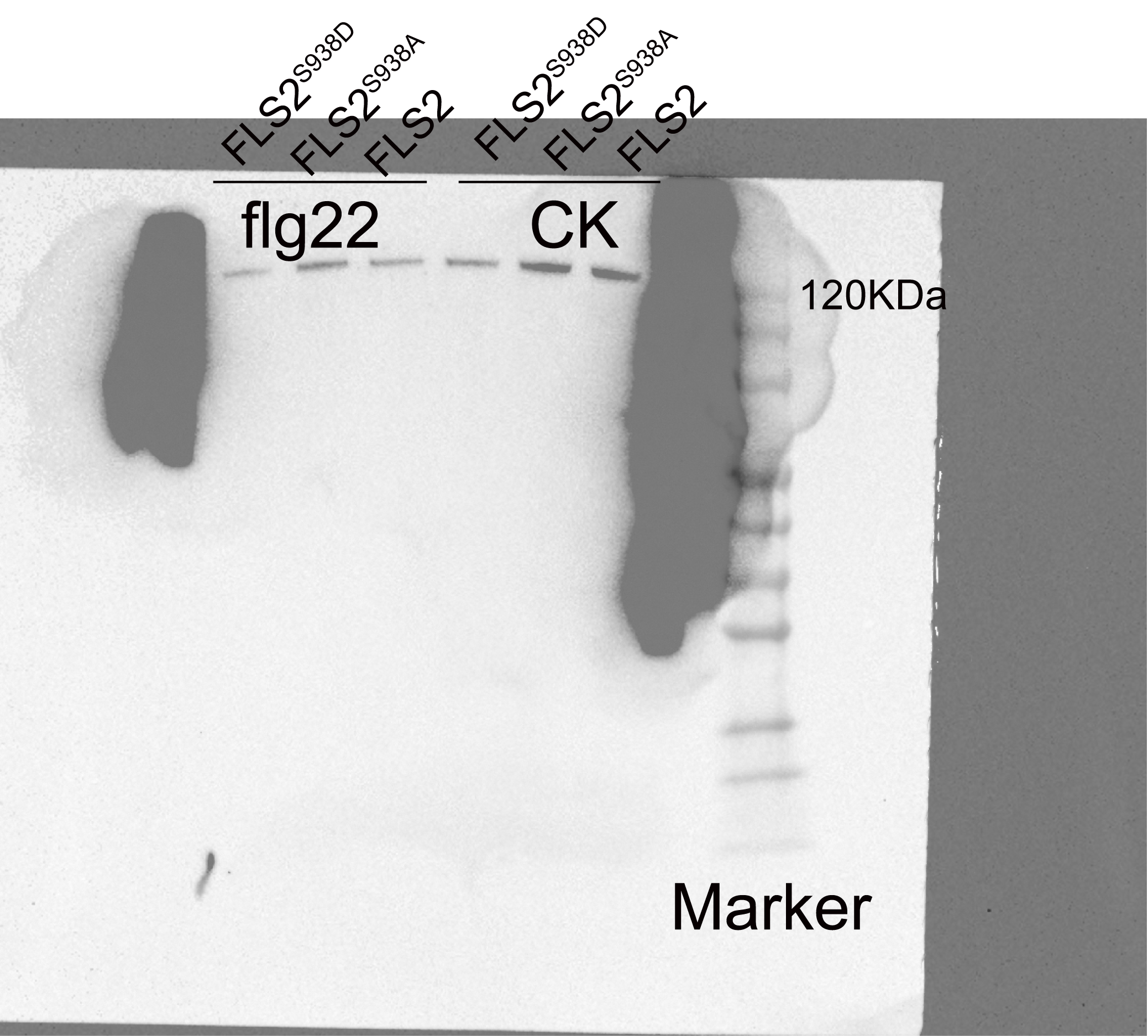

Supplement: Figure 3—source data 6. [file elife-91072-fig3-data6.zip › Figure 3—source data 6/Figure3E---SourceData2.tif]

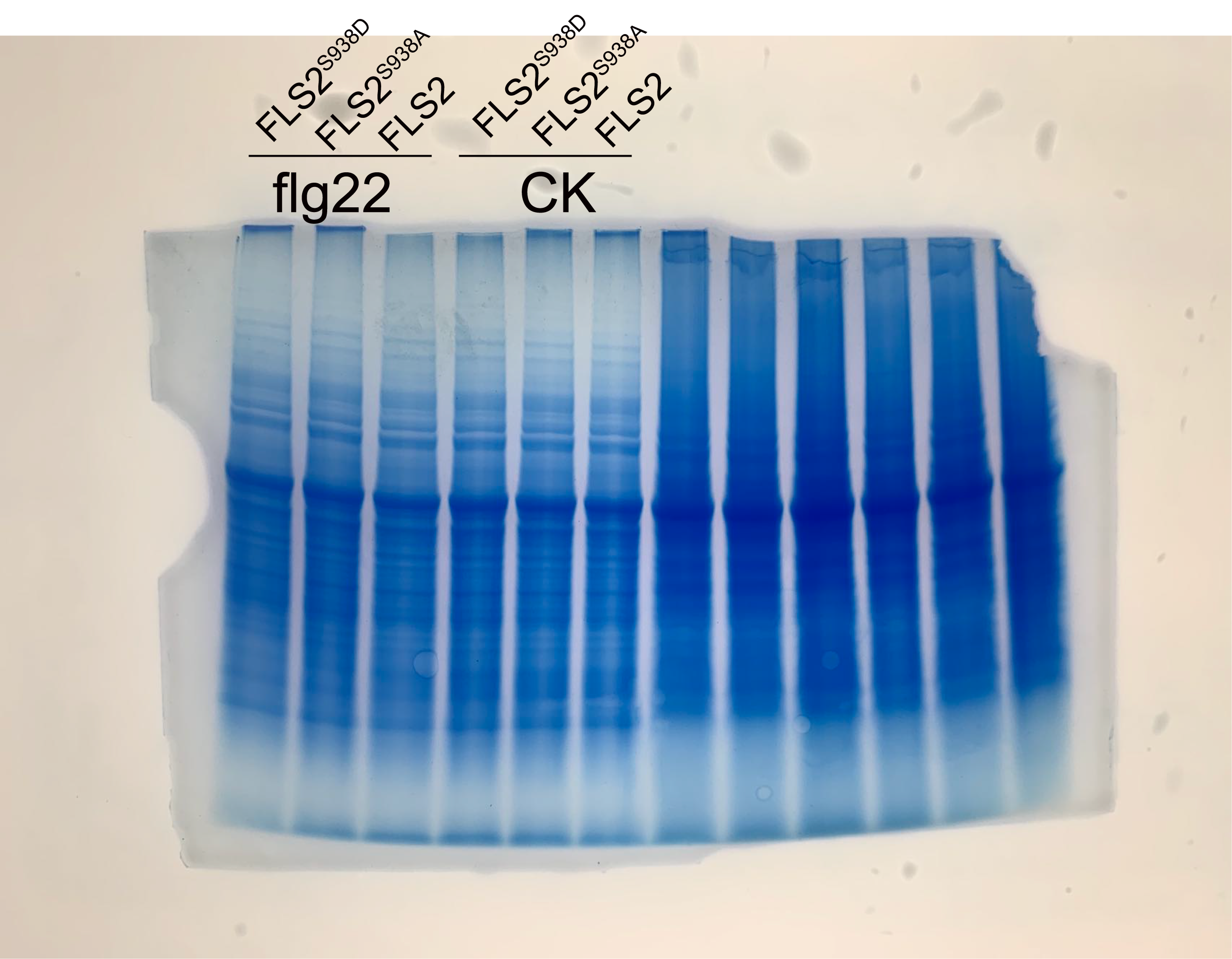

Supplement: Figure 3—source data 6. [file elife-91072-fig3-data6.zip › Figure 3—source data 6/Figure3E---SourceData3.tif]

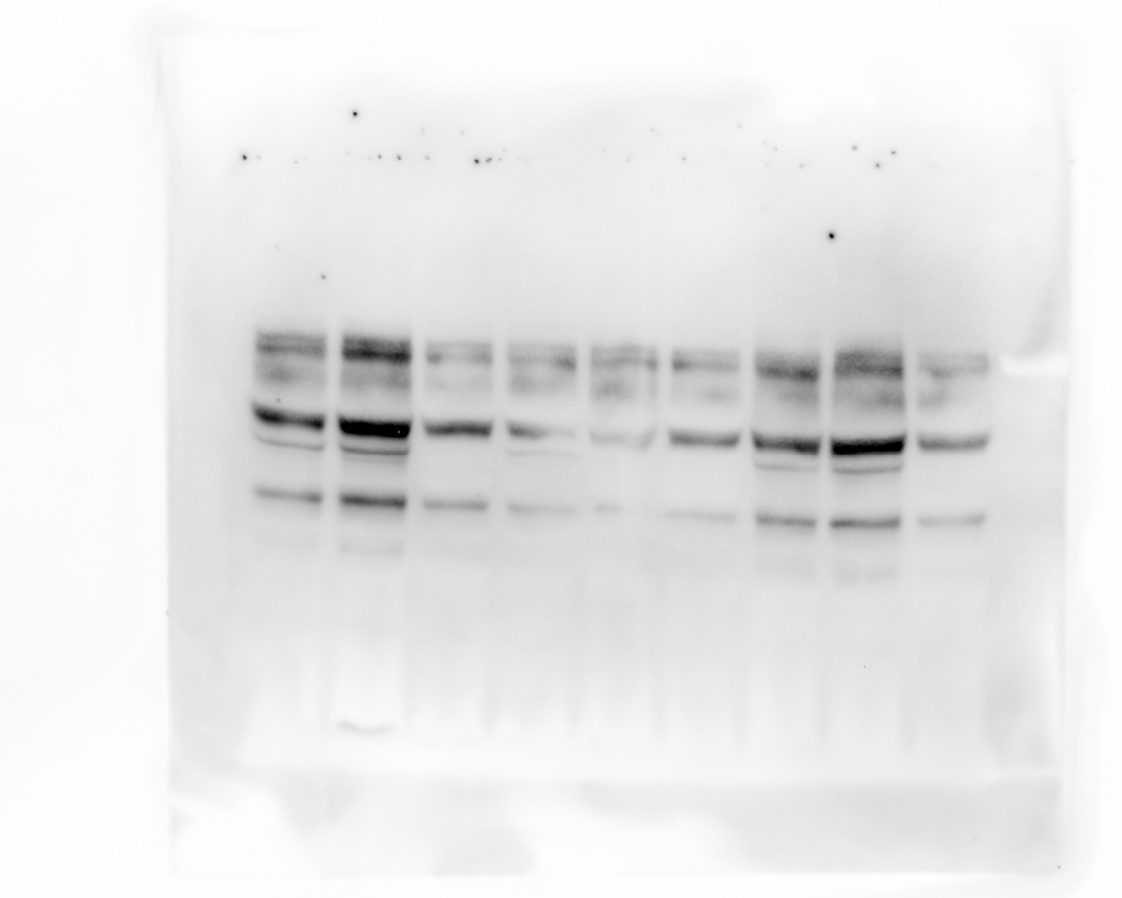

Supplement: Figure 4—figure supplement 1—source data 1. [file elife-91072-fig4-figsupp1-data1.zip › Figure 4 - figure supplement-source data 1/Figure 4 - figure supplement 1---SourceData 1.jpg]

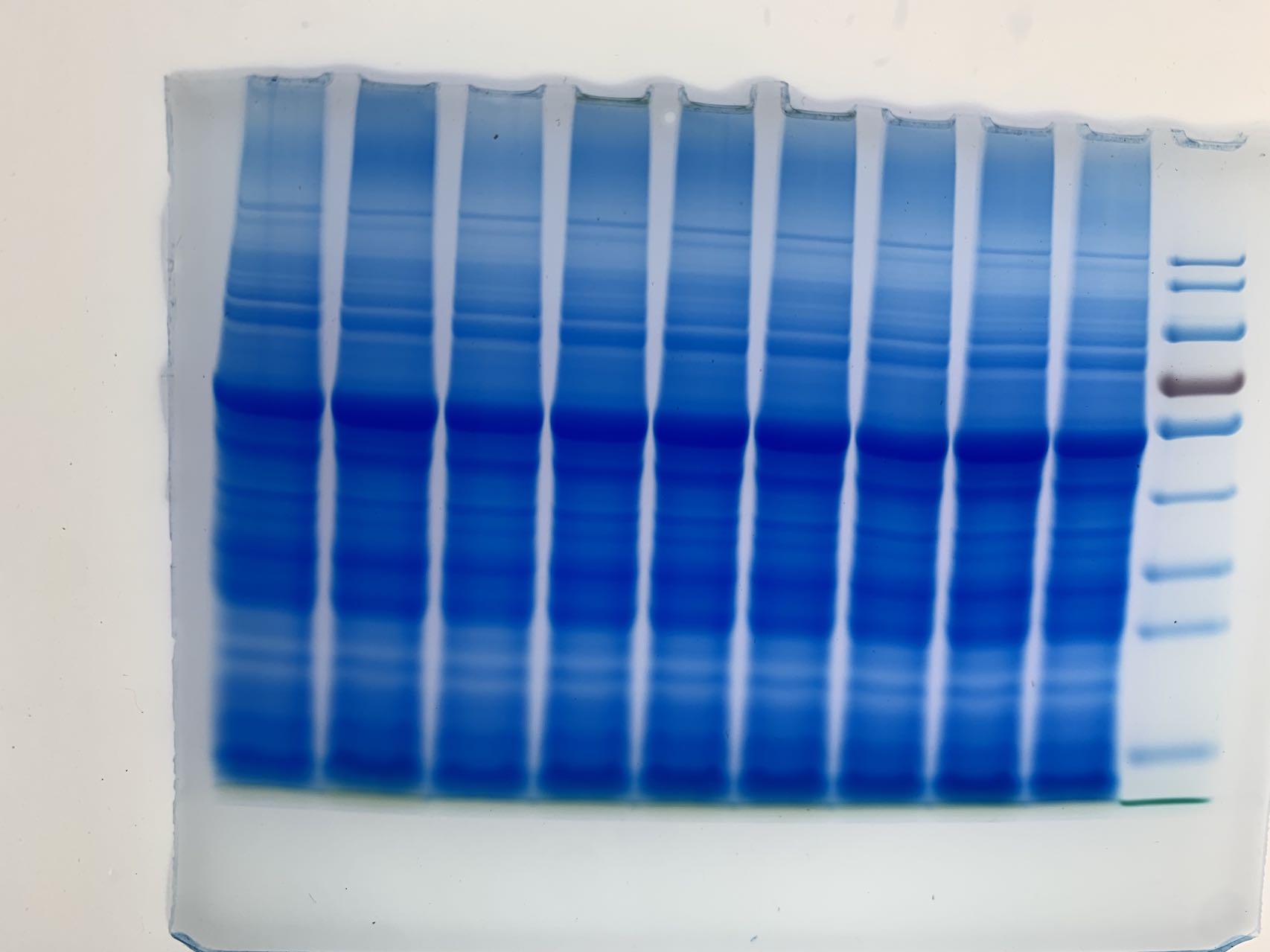

Supplement: Figure 4—figure supplement 1—source data 1. [file elife-91072-fig4-figsupp1-data1.zip › Figure 4 - figure supplement-source data 1/Figure 4 - figure supplement 1---SourceData 2.jpg]

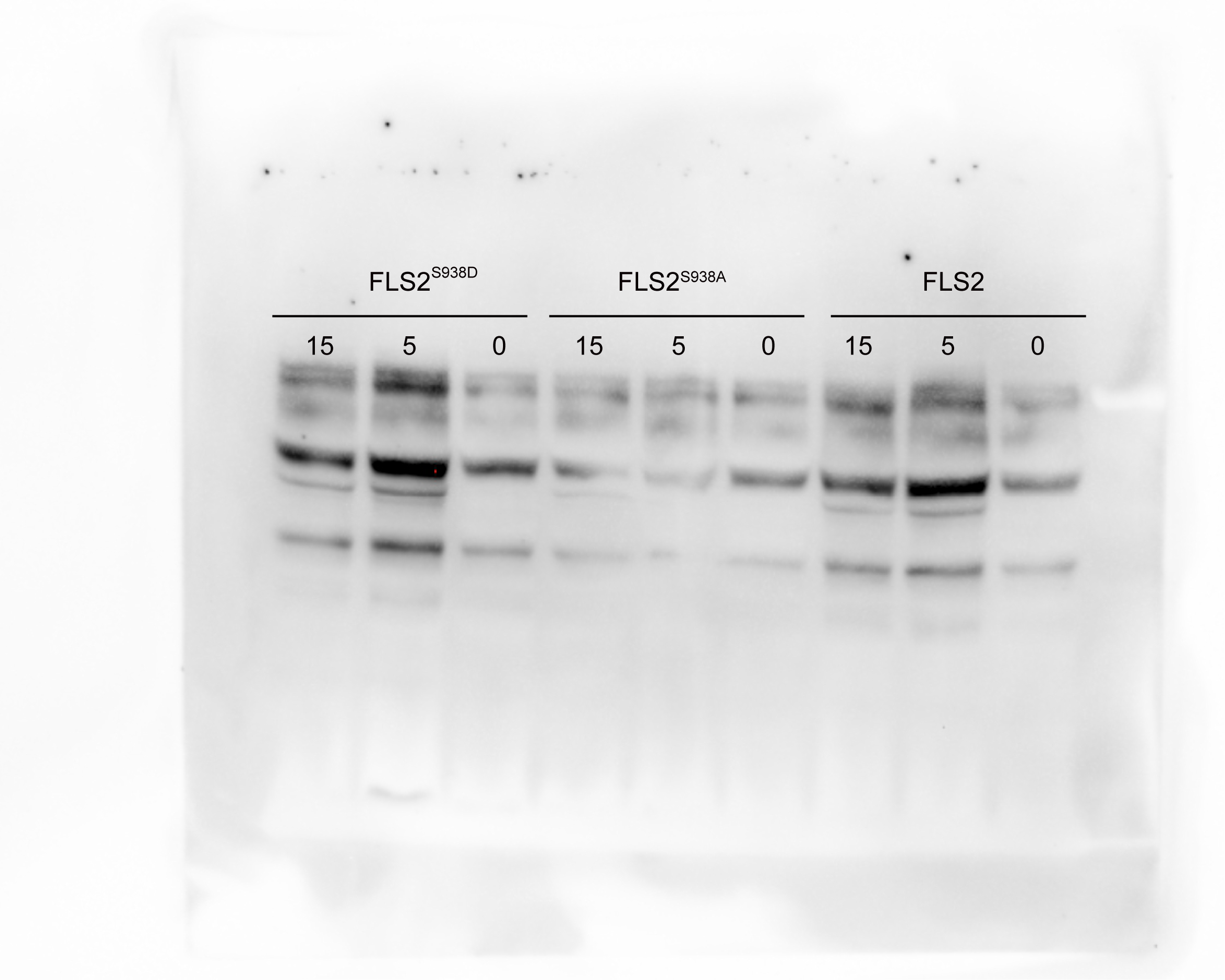

Supplement: Figure 4—figure supplement 1—source data 2. [file elife-91072-fig4-figsupp1-data2.zip › Figure 4 - figure supplement-source data 2/Figure 4 - figure supplement 1-source data 1.tif]

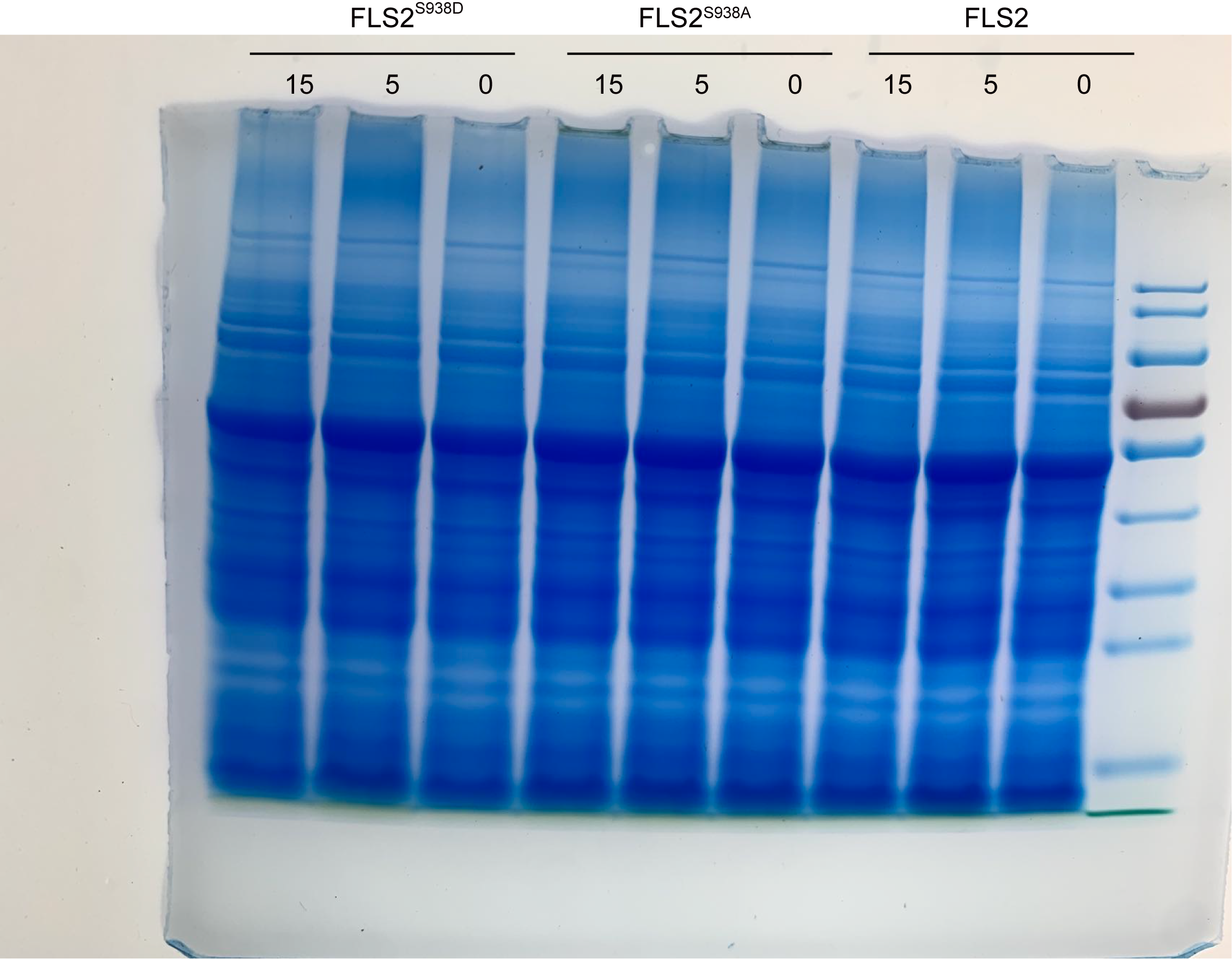

Supplement: Figure 4—figure supplement 1—source data 2. [file elife-91072-fig4-figsupp1-data2.zip › Figure 4 - figure supplement-source data 2/Figure 4 - figure supplement 1-source data 2.tif]

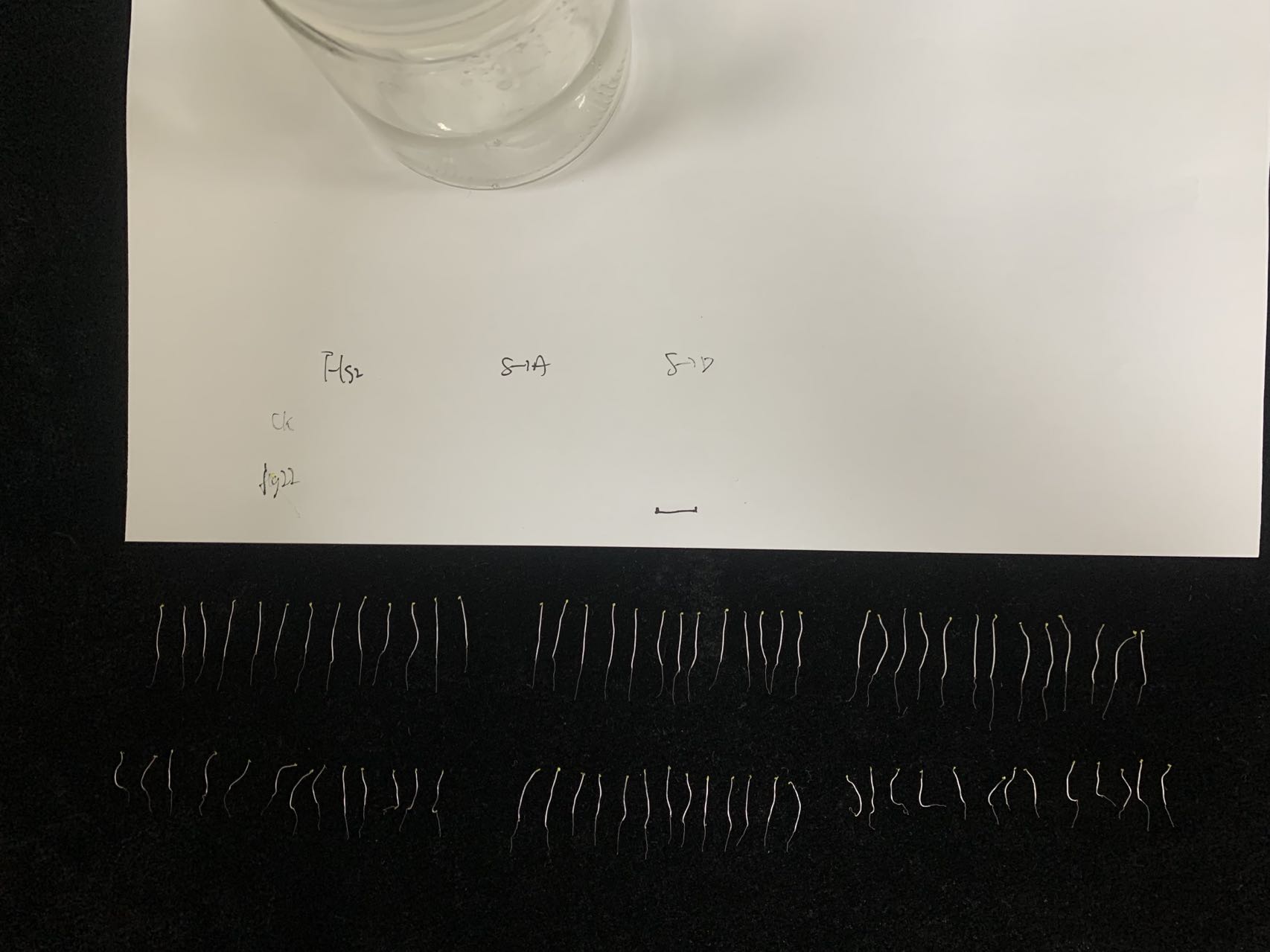

Supplement: Figure 4—figure supplement 3—source data 1. [file elife-91072-fig4-figsupp3-data1.zip › Figure 4 - figure supplement-source data 4/Figure 4 - figure supplement 3-source data .jpg]
